# Affect and Mental Health Across the Lifespan During a Year of the COVID-19 Pandemic: The Role of Emotion Regulation Strategies and Mental Flexibility

**DOI:** 10.1037/emo0001238

**Published:** 2023-05-18

**Authors:** Savannah Minihan, Annabel Songco, Elaine Fox, Cecile D. Ladouceur, Louise Mewton, Michelle Moulds, Jennifer H. Pfeifer, Anne-Laura Van Harmelen, Susanne Schweizer

**Affiliations:** 1 School of Psychology, University of New South Wales; 2 School of Psychology, University of Adelaide; 3 Department of Psychology, University of Pittsburgh; 4 Centre for Healthy Brain Ageing, University of New South Wales; 5 Department of Psychology, University of Oregon; 6 Brain, Safety and Resilience, Education and Child Studies, Leiden University; 7 Department of Psychology, University of Cambridge

**Keywords:** COVID-19, mental health, affect, emotion regulation, mental flexibility

## Abstract

During the COVID-19 pandemic, there has been a rise in common mental health problems compared to prepandemic levels, especially in young people. Understanding the factors that place young people at risk is critical to guide the response to increased mental health problems. Here we examine whether age-related differences in mental flexibility and frequency of use of emotion regulation strategies partially account for the poorer affect and increased mental health problems reported by younger people during the pandemic. Participants (*N* = 2,367; 11–100 years) from Australia, the UK, and US were surveyed thrice at 3-month intervals between May 2020 and April 2021. Participants completed measures of emotion regulation, mental flexibility, affect, and mental health. Younger age was associated with less positive (*b* = 0.008, *p* < .001) and more negative (*b* = −0.015, *p* < .001) affect across the first year of the pandemic. Maladaptive emotion regulation partially accounted for age-related variance in negative affect (β = −0.013, *p* = .020), whereby younger age was associated with more frequent use of maladaptive emotion regulation strategies, which, in turn, was associated with more negative affect at our third assessment point. More frequent use of adaptive emotion regulation strategies, and in turn, changes in negative affect from our first to our third assessment, partially accounted for age-related variance in mental health problems (β = 0.007, *p* = .023). Our findings add to the growing literature demonstrating the vulnerability of younger people during the COVID-19 pandemic and suggest that emotion regulation may be a promising target for intervention.

With the emergence of the COVID-19 pandemic and the subsequent implementation of sweeping restrictions to daily life, researchers quickly warned of an impending mental health crisis (Holmes et al., 2020). Concerns were raised, in particular, for adolescent mental health (Orben et al., 2020), with meta-analytic evidence showing a significant effect of the pandemic on youth mental health (Racine et al., 2021). Adolescence (10–24 years; Sawyer et al., 2018) is a vulnerable period for the emergence of mental health problems, in particular, emotional disorders (e.g., depression and anxiety; Rapee et al., 2019). This vulnerability has been attributed, in part, to still-developing emotion regulation (Ahmed et al., 2015; Silvers, 2022). Poor emotion regulation is a key transdiagnostic risk factor in the development of mental health disorders, including depression and anxiety, in both adolescence and adulthood (Aldao et al., 2010; Schäfer et al., 2017). Here we examine whether age-related differences in the use of emotion regulation strategies and mental flexibility partially account for the poorer affect and higher levels of mental health problems reported by younger relative to older people during the first year of the pandemic.

Emotion regulation, defined as the monitoring, evaluation, and modification of one’s emotional reaction in order to accomplish one’s goals (Thompson, 1994), is a critical skill for successfully navigating novel, stressful, or affectively charged situations. Emotion regulation strategies are often conceptualized as adaptive or maladaptive depending on their association with affective experiences and mental health. For example, research has shown that emotion regulation strategies such as suppression, avoidance, and rumination can increase negative and reduce positive affect in the long-term (Aldao et al., 2010; Boemo et al., 2022; Gross & John, 2003; Joormann & Vanderlind, 2014; Moberly & Watkins, 2008; Nezlek & Kuppens, 2008) and have been found in meta-analyses to be associated with more mental health problems in both adolescents and adults (Aldao et al., 2010; Schäfer et al., 2017). Conversely, putatively adaptive strategies such as reappraisal, acceptance, and problem solving are associated with fewer mental health problems across the lifespan (Aldao et al., 2010; Schäfer et al., 2017).

Critically, our capacity to regulate our emotions effectively and implement adaptive emotion regulation strategies improves from early life, through adolescence, to adulthood, in parallel with the protracted development of the neural substrates underlying effective emotion regulation (Ahmed et al., 2015; McRae et al., 2012; Silvers et al., 2012; Zimmermann & Iwanski, 2014). In studies of young people, there is evidence to suggest that early adolescence may involve a shift toward the use of more maladaptive (Gullone et al., 2010) and less adaptive strategies (Cracco et al., 2017). For example, in a cross-sectional study of children and adolescents aged between 8 and 18 years old, Cracco et al. (2017) observed reduced use of adaptive strategies (i.e., problem solving, distraction, forgetting, acceptance, humor enhancement, cognitive problem solving, and revaluation) and increased use of maladaptive strategies (i.e., giving up, withdrawal, rumination, self-devaluation, and aggressive actions) in 12–15-year-olds compared with younger and older participants. Research on the transition from adolescence to adulthood has found linear increases in the use of adaptive regulation strategies (Zimmermann & Iwanski, 2014) as well as improvements in emotion regulation capacity (Silvers et al., 2012). For example, among participants aged 11–50 years, the use of adaptive emotion regulation strategies (e.g., “I calm down first and then deal with the situation again”; “I concentrate on what to do next”) increased linearly with age (Zimmermann & Iwanski, 2014). Together these findings suggest that adolescents are more likely to deploy maladaptive emotion regulation strategies and less likely to use adaptive strategies compared to both children and adults.

Beyond adolescence and middle adulthood, emotion regulation appears to improve again in older adulthood. Improved emotion regulation and emotional well-being in older age have been proposed to be accounted for by heightened awareness and acceptance of shrinking time horizons in this age group, which prompts greater allocation of resources to emotion regulation and more effective implementation of adaptive emotion regulation strategies (see socioemotional selectivity theory; Carstensen et al., 2003). In line with the socioemotional selectivity theory, older adults report greater perceived control over their emotions (Gross et al., 1997; Phillips et al., 2006), and some studies show greater use of adaptive (e.g., reappraisal) and less use of maladaptive (e.g., suppression) strategies in older age (e.g., John & Gross, 2004; Schirda et al., 2016; however see: Eldesouky & English, 2018). Such age advantages in affective experiences have been shown to persist amidst the COVID-19 pandemic, which posed a particular threat to the physical health and well-being of older individuals. For example, in a study of 18–76-year-olds, older age was associated with less frequent and intense negative emotions and more frequent and intense positive emotions, even while controlling for risk of contracting COVID-19 and risk of COVID-19-related complications (Carstensen et al., 2020). Similarly, in a study of younger (18–25 years) and older (55–79 years) individuals, older individuals reported less negative affect when dealing with stress related to the pandemic, as well as more problem-focused regulation strategies (incl. active coping and planning), and less socially supported (emotional social support, instrumental social support, venting) and avoidant (incl. behavioral and mental disengagement) regulation strategies (Young et al., 2021).

Differential use of emotion regulation strategies across the lifespan, then, may have contributed to differences in affect and mental health during the pandemic. In particular, more frequent use of maladaptive and less frequent use of adaptive emotion regulation strategies in younger people compared to adults and older adults may have contributed to the increased negative affectivity, reduced positive affectivity, and heightened mental health problems observed in adolescents during the pandemic.

Indeed, several studies have implicated both trait emotion regulation ability and the use of specific emotion regulation strategies in adolescent affective responding during the pandemic. Specifically, poorer pre-COVID-19 emotion regulation abilities predicted greater increases in mental health problems among adolescents aged 15–17 years (Breaux et al., 2021). Similarly, in adolescents aged 10–15 years, Weissman et al. (2021) observed increased internalizing symptoms early in the pandemic among youth who reported greater rumination before the pandemic and those who reported greater suppression and lower reappraisal early in the pandemic. Conversely, Romm et al. (2021) found that reappraisal and suppression use prior to the pandemic did not predict change in psychosocial outcomes from before to during the pandemic among adolescents aged 14–16 years. The mixed findings on the association between prepotent emotion regulation tendencies and mental health during the pandemic may be in part accounted for by the fact that these trait measures do not capture flexibility in the application of context-appropriate regulatory strategies.

The efficacy of regulatory strategies depends on the situational context (Aldao, 2013; Bonanno & Burton, 2013). That is, individuals need to flexibly select and implement a range of emotion regulation strategies depending on varying contextual demands. In line with this, a cross-sectional study of adolescents aged 12­–17 years found that adolescents who relied on few emotion regulation strategies reported more symptoms of depression and anxiety, compared to those adolescents with a broader repertoire of emotion regulation strategies (Lougheed & Hollenstein, 2012). In adults, flexible use of acceptance and problem solving was associated with less mental health problems (Aldao & Nolen-Hoeksema, 2012), and greater emotion regulation flexibility was associated with more positive and less negative affect (Battaglini et al., 2022). Few studies, however, tend to dissociate the *frequency* with which maladaptive and adaptive strategies are implemented, and the *flexibility* with which such strategies are used across varying contexts (Silvers & Guassi Moreira, 2019). Indeed, mental flexibility, more broadly, characterized by an ability to recognize, adapt, and shift strategies in response to various situational demands has been shown to be a fundamental aspect of mental health and well-being across the lifespan (Kashdan & Rottenberg, 2010) and has been associated with flexible use of a range of emotion regulation strategies (Kashdan et al., 2020). Age-related differences in affect and mental health problems during the pandemic, then, may be partially accounted for by both age-related differences in the use of emotion regulation strategies as well as differences in mental flexibility.

## The Present Study

The present study aimed to examine whether differences in the deployment of (mal)adaptive emotion regulation strategies and mental flexibility would account for age-related differences in affect and mental health during the first year of the pandemic. Specifically, the present study investigated the relationship between both maladaptive and adaptive strategy use and mental flexibility with affective experiences and mental health problems across a year of the COVID-19 pandemic.

We investigated this research question using data from the COVID-19 Risks Across the Lifespan (CORAL) Study. The CORAL study is an online longitudinal study, in which participants from early adolescence to old age (11–100 years) were assessed at three 3-monthly intervals between May 2020 (T1) and April 2021 (T3). The CORAL study included measures assessing the frequency of use of emotion regulation strategies, mental flexibility, negative and positive affect, and mental health problems. This data allowed us to investigate age-related differences in the relationship between use of emotion regulation strategies, mental flexibility, negative and positive affect, and mental health problems during 1 year of the COVID-19 pandemic.

Our hypotheses preregistered (https://osf.io/49gqh) prior to analyses were that: younger age would be associated with less positive and more negative affect across time (H1). Given the age-related differences in the habitual use of emotion regulation strategies reviewed above, as well as the association between emotion regulation and affective experiences, we next investigated whether the use of specific (mal)adaptive emotion regulation strategies and mental flexibility accounted for any age-related differences in average levels of affect. We predicted that the association between age and positive and negative affect would be partially accounted for by the frequency of use of emotion regulation strategies and mental flexibility. That is, emotion regulation strategies and mental flexibility measured at T1 would partially account for variance in the relationship between age and positive and negative affect at T3 (H2). Habitual use of emotion regulation strategies has been shown to drive changes in affect across time, with increases in negative affect and decreases in positive affect being hallmark symptoms of emotional disorders. Therefore, we next investigated whether the frequency of use of emotion regulation strategies and mental flexibility, and their effects on change in negative and positive affect, accounted for any age-related differences in mental health problems. We predicted that emotion regulation strategies and mental flexibility measured at T1 and their effect on changes in positive and negative affect (i.e., from T1 to T3) would partially account for variance in the relationship between age and mental health problems at T3 (H3).

## Method

### Participants

The CORAL study collected a convenience sample of 3,208 participants who consented to and were eligible to participate. Participants were recruited via various means, including advertisements on social media and school newsletters, registration of the study on online research participation platforms (e.g., MQ participate platform, SANE Australia Forum, COVID Minds, NIHR, Children Helping Science, and Assessment of COVID-19 Experiences for Adolescents), emailing the study information to relevant organizations (e.g., general and youth mental health organizations, pregnancy and parenting organizations), and by word of mouth. At each timepoint, participants had the chance to win an AUD $100 (£50 OR US$60) gift voucher. Participants received an AUD $10 gift voucher at each subsequent timepoint (T2 and/or T3), in addition to being entered in the draw for the AUD $100 voucher.

In order to take part in the CORAL study, participants were required to (a) be residing in Australia, the United Kingdom, or the United States, (b) be aged 11 years or older, (c) be fluent in English, (d) have no history of neurodevelopmental or neurological disorder, (e) have no history of traumatic brain injury, and (e) be capable of providing informed consent. Additionally, participants were excluded from the current study if they had more than 20% missing data on the mental health outcomes at T1 (*n* = 841), had additional duplicate records in the dataset (T1: *n* = 9, T2: *n* = 21, T3: *n* = 13), or responded incorrectly to more than one attention check item (T1: *n* = 8, T3: *n* = 1). This resulted in a sample of 2,367 participants, aged 11–100 years (*M* [*SD*]_age__=_ 38.85 [16.94] years). The majority of participants identified as female, White, and of high socioeconomic status (Table 1). See Minihan et al. (2022) for attrition analyses as a function of demographic characteristics.[Table tbl1]

### Measures

Only those measures included in the analyses for the current study are reported below (see Minihan et al., 2022 for a complete list of all measures included in the CORAL study).

#### Demographics

Participant demographics were assessed at T1 and included age, self-identified gender, self-identified ethnicity, country of residence, and highest educational attainment, as a proxy for socioeconomic status. For participants under the age of 18, the average of their parent’s/guardian’s highest educational attainment was used.

#### Affect

To assess positive and negative affect, participants indicated the extent to which they had experienced various positive (i.e., content, happy, relieved, calm, appreciative) and negative (i.e., anxious, angry, afraid, sad, worried, irritable, concerned, stressed, distressed, lonely, bored, hopeless, frustrated, disappointed) emotions in the previous 2 weeks because of the COVID-19 outbreak and resulting changes to daily life. Responses were provided on a six-point scale, ranging from 1 (*Not at all*) to 6 (*Extremely*) and collected at all timepoints. Average positive and negative affect at each time point was computed by averaging the respective items and a change score was calculated by subtracting average positive/negative affect at T1 from average positive/negative affect at T3. The positive and negative affect items demonstrated acceptable internal consistency at all timepoints (positive affect: Revell’s total omega (ω*T*) ranged from .85 to .87; negative affect: ω*T* ranged from .95 to .96).

#### Emotion Regulation Strategies

Participants responded to a series of bespoke items at T1 assessing the frequency with which they tried to influence their emotions by using maladaptive strategies: suppression (“keeping my emotions to myself”), avoidance (“avoiding a situation”), and rumination (“focusing attention on my emotions and problems”), and adaptive strategies: acceptance (“accepting my emotions, even when negative”), distraction (“distracting myself from a situation”), reappraisal (“changing the way I thought about a situation”), problem solving (“taking action to change a situation”), and social support (“turning to others for support”) over the previous 2 weeks (Lennarz et al., 2019). A single item assessed each strategy and participants responded to each item on a seven-point Likert scale, ranging from 0 (*Not at all*) to 6 (*Very frequently*). Acceptable internal consistency for these items was observed (ω*T* = .75).

In line with conceptualizations of worry as a cognitive emotion regulation strategy (Mennin et al., 2002), we also included the Penn State Worry Questionnaire (PSWQ), which assesses trait worry. In the current study, the 14-item youth version was used (PSWQ-C; Chorpita et al., 1997), due to the wide age range of participants. Participants indicated the extent to which such items as “My worries really bother me” were true of themselves on a 4-point Likert scale, ranging from 0 (*Never true*) to 3 (*Always true*). The questionnaire has demonstrated good psychometric properties, including good reliability and convergent and discriminant validity, in both community and clinical samples (Chorpita et al., 1997). In the current study, good internal consistency was observed (ω*T* *=* .96).

#### Mental Flexibility

Mental flexibility was assessed with the Mental Flexibility Questionnaire (MFQ). The MFQ was developed by the Oxford Centre for Emotions and Affective Neuroscience and indexes psychological flexibility, that is, the capacity to adapt and shift one’s perspective and strategies in order to deal with problems (Parsons et al., 2022). In the current study, the eight-item version was used. In relation to the past week, participants indicated the extent to which they agreed with such items as, “I have been good at adapting to different situations” on a 6-point Likert scale, ranging from 1 (*Strongly disagree*) to 6 (*Strongly agree*). The MFQ demonstrated good internal consistency in the current study (ω*T* = .92).

#### Mental Health Problems

In the current study, mental health problems were operationalized as depressive and anxiety symptoms and mental well-being and measured at all timepoints. Depressive symptoms were assessed with the eight-item Patient Health Questionnaire (PHQ-8; Kroenke et al., 2001), which has previously demonstrated good psychometric properties (Beard et al., 2016; Martin et al., 2006). In relation to the previous 2 weeks, participants indicated the extent to which they had been bothered by such things as “Little interest or pleasure in doing things” on a 4-point Likert scale, ranging from 0 (*Not at all*) to 3 (*Nearly every day*). Good internal consistency was observed in the current study (ω*T* ranged from .93 to .94).

The seven-item General Anxiety Disorder scale (GAD-7; Spitzer et al., 2006) assessed anxiety symptoms. Participants indicated how often they had been bothered by such problems as “Feeling nervous, anxious, or on edge” over the previous 2 weeks on a 4-point Likert scale, ranging from 0 (*Not at all*) to 3 (*Nearly every day*). The GAD-7 has been shown to have good reliability and validity (Beard & Björgvinsson, 2014; Löwe et al., 2008), and good internal reliability was observed in the current study (ω*T* ranged from .95 to .96).

Mental well-being was assessed with the seven-item Warwick-Edinburgh Mental Wellbeing Scale (WEMWBS; Stewart-Brown et al., 2009), which has demonstrated good psychometric properties (Stewart-Brown et al., 2009). Participants indicated the extent to which such items as “I’ve been feeling optimistic about the future,” described their experience over the previous 2 weeks, on a 5-point Likert scale, ranging from 1 (*None of the time*) to 5 (*All of the time*). This measure was reverse scored for analyses. Acceptable internal reliability was observed in the current study (ω*T* ranged from .89 to .91).

#### COVID-19 Risk

A series of bespoke items were included to assess COVID-19-related exposure and health risk. Participants indicated whether they had ever been tested for COVID-19; whether they or anyone in their house had ever been quarantined due to possibly having COVID-19; whether they had been hospitalized due to COVID-19; or whether they knew anyone personally who had been diagnosed with, hospitalized due to, or passed away from COVID-19. A weighted composite score was computed (see the online supplementary material 1 for further details) and included in analyses as a covariate, to control for the potential impact of COVID-19-related exposure and health risk on mental health problems (Hossain et al., 2020).

### Procedure

All participants provided online informed consent prior to taking part in the study. Parental consent via an online consent form was additionally obtained for participants under 18 years. Following the provision of parental consent, parents were provided with a link and study access code for their children. Participants completed the online measures via Qualtrics (Qualtrics, Provo, UT) in the following order: demographics, COVID-19 risk, positive and negative affect, mental health problems, mental flexibility, and frequency of use of emotion regulation strategies. This study was approved by the University of New South Wales Human Research Ethics Committee (HC200287).

### Data Analysis

Prior to hypothesis testing, a confirmatory factor analysis was conducted to validate the factor structure of the emotion regulation and mental flexibility items. Our preregistered method was to fit a measurement model with three latent variables: one indexing maladaptive emotion regulation strategies; one indexing adaptive emotion regulation strategies; and one indexing mental flexibility. We proposed that the following items would load positively on the maladaptive emotion regulation factor: “keeping my emotions to myself” (suppression), “avoiding a situation” (avoidance), “focusing attention on my emotions and problems” (rumination), and all items from the PSWQ-C. However, during the peer review process, it was highlighted that there was substantial conceptual overlap between items from the PSWQ-C and the GAD-7, as well as items from our affect measures (i.e., worry was included as a negative affect item). Such conceptual overlap between variables may be driving any significant results observed; consequently, the items from the PSWQ-C were not included in the analyses, which represents a deviation from our pre-registered protocol (for analyses as per our pre-registration, please see Tables 13–16 in the online supplemental materials). For the adaptive emotion regulation factor, the following items were proposed to load positively: “changing the way I thought about a situation” (reappraisal), “accepting my emotions, even when negative” (acceptance), “turning to others for support” (social support), “distracting myself from a situation” (distraction), and “taking action to change a situation” (problem solving). Finally, we proposed that all items from the MFQ would load positively on the mental flexibility factor. As in Minihan et al. (2022), mental health problems were modeled as a higher-order latent variable, with the PHQ-8 items, GAD-7 items, and reverse-coded WEMWBS items loaded onto individual factors, with these three factors then loaded onto an overall mental health general factor.

The first hypothesis, that younger age would be associated with less positive and more negative affect across time, was tested with a linear mixed effects model, separately for positive and negative affect. In this model, age, timepoint, and their interaction were included as fixed effects, and positive/negative affect as the outcome variable. Gender (dummy coded as female = 1, other = 0), country of residence (dummy coded with Australia as the reference level), ethnicity (dummy coded as White = 1, other = 0), and COVID-19 risk were included as covariates. Timepoint was modeled as a continuous variable (with the first timepoint coded as 0) and age and COVID-19 risk were mean centered. Participant ID was included as a random effect. As per our preregistration, age was modeled as a continuous variable; however, given our particular interest in adolescence, we also investigated the change in affect as a function of age group (adolescents: 11–24 years; adults: 25–64 years; older adults: 65+ years).

To examine our second hypothesis, that emotion regulation strategies and mental flexibility measured at T1 would partially account for variance in the relationship between age and positive/negative affect at T3, a mediation model was specified, separately for positive and negative affect. Age was included as the predictor variable, the emotion regulation and mental flexibility latent variables as multiple mediators, and positive/negative affect at T3 as the outcome variable. Gender, country of residence, ethnicity, COVID-19 risk, and positive/negative affect at T1 were included as covariates, and the emotion regulation and mental flexibility latent variables were allowed to covary.

Finally, to investigate our third hypothesis, that emotion regulation strategies and mental flexibility, and their effect on changes in positive/negative affect from T1 to T3 would partially account for variance in the relationship between age and mental health problems at T3, an additional mediation model was specified, separately for positive and negative affect. Again, age was included as the predictor variable, the emotion regulation and mental flexibility latent variables as multiple mediators, change in positive/negative affect as a serial mediator, and mental health problems at T3 as the outcome variable. Gender, country of residence, ethnicity, COVID-19 risk, and mental health problems at T1 were included as covariates, and the emotion regulation and mental flexibility latent variables were allowed to covary, as were the error variances of the repeated mental health measures. The pattern of results for our second and third hypotheses remained consistent when age group was included as the predictor variable. However, as per our preregistration, we only report the results for age modeled as a continuous predictor here.

We opted to analyze data from T1 and T3, as we expected the impact of emotion regulation and mental flexibility on affect and the impact of changes in affect on mental health problems to be more likely to show effects across a six- rather than 3-month follow-up period. However, following recommendations arising during peer review, we have included the exploratory analyses, including T2 affect and T2 mental health problems in the supplementary materials (see Tables 6, 7, 11, and 12 in the online supplemental materials).

Robust full information maximum likelihood estimation was used to account for missing data. Model fit was assessed using standard criteria, with acceptable fit indicated by comparative fit index (CFI) and Tucker–Lewis index (TLI) values ≥.90, and root mean square error of approximation (RMSEA) and standardized root mean square residual (SRMR) values ≤.08 (Schermelleh-Engel et al., 2003). A Bonferroni-corrected α level of .025 was applied to all analyses, to account for running all analyses twice (i.e., once for positive and once for negative affect).

### Transparency and Openness

We report how we determined our sample size, all data exclusions, all manipulations, and all measures in the pre-registered (https://osf.io/49gqh) study. All analyses were conducted in R Studio version 4.1.2 using the *psych* package (Revelle, 2021), the *tidyverse* package (Wickham et al., 2019), the *lavaan* package (Rosseel, 2012), the *qwraps2* package (DeWitt & Bennett, 2021), the *afex* package (Singmann et al., 2021), the *reshape2* package (Wickham, 2007), the *sjPlot* package (Lüdecke, 2022), the *apaTables* package (Stanley, 2022), and the *emmeans* package (Lenth et al., 2023). Upon publication, all analyses will be made available on the Open Science Framework (https://osf.io/r5chj/).

## Results

### Preliminary Analyses

A confirmatory factor analysis demonstrated an acceptable fit for the emotion regulation strategies and mental flexibility measurement model (CFI = .886, TLI = .864, RMSEA = .085, SRMR = .082). All items loaded significantly and positively on their respective factors. The three-factor measurement model was thus retained for analyses, with one factor indexing maladaptive emotion regulation strategies (incl. suppression, avoidance, and rumination), one indexing adaptive emotion regulation strategies (incl. reappraisal, acceptance, social support, distraction, and problem solving), and one indexing mental flexibility (incl. all items from the MFQ). The higher-order mental health measurement model also demonstrated a good fit to the data (CFI = .915, TLI = .905, RMSEA = .081, SRMR = .043) and is described further in Minihan et al. (2022). For descriptives and correlations between all variables, see Table 1 in the online supplemental materials.

### The Relationship Between Age and Affect Across 1 Year of the COVID-19 Pandemic

To address H1, that younger age would be associated with less positive and more negative affect across time, a linear mixed effects model was specified, separately for positive and negative affect (Table 2; for results without covariates, see Table 2 in the online supplemental materials). While there was no significant change in positive affect across time, there was a significant decrease in negative affect across time. For both positive and negative affect, there was a main effect of age, with younger age being associated with less positive affect and more negative affect. The effects of time and age did not interact; that is, change in affect across time did not differ as a function of age. In exploratory analyses including age group as the predictor variable, the main effect of age group was significant for both positive and negative affect (positive affect model: *F* = 22.576, *p* < .001; negative affect model: *F* = 68.725, *p* < .001), and the interaction between age group and affect was not significant (positive affect model: *F* = 1.927, *p* = .146; negative affect model: *F* = 1.988, *p* = .137). Pairwise comparisons with Bonferroni correction showed that, collapsed across time, positive affect was significantly lower in both adolescents (*b* = −0.536, *SE* = 0.070, 95% CI [−0.702 to −0.369], *p* < .001) and adults (*b* = −0.395, *SE* = 0.056, [−0.528 to −0.262], *p* < .001) compared to older adults. The pairwise comparison between adolescent and adult positive affect was not significant according to our overall Bonferroni correction (*b* = −0.141, *SE* = 0.054, [−0.271 to −0.012], *p* = .027). Negative affect was significantly greater in adolescents compared to both adults (*b* = 0.360, *SE* = 0.058, [0.219–0.501], *p* < .001) and older adults (*b* = 0.873, *SE* = 0.076, [0.691–1.055], *p* < .001). Negative affect was also significantly greater in adults compared to older adults (*b* = 0.513, *SE* = 0.061, [0.366–0.659], *p* < .001).[Table tbl2]

### The Relationship Between Age, Emotion Regulation Strategies, Mental Flexibility, and Affect

**Figure 1 fig1:**
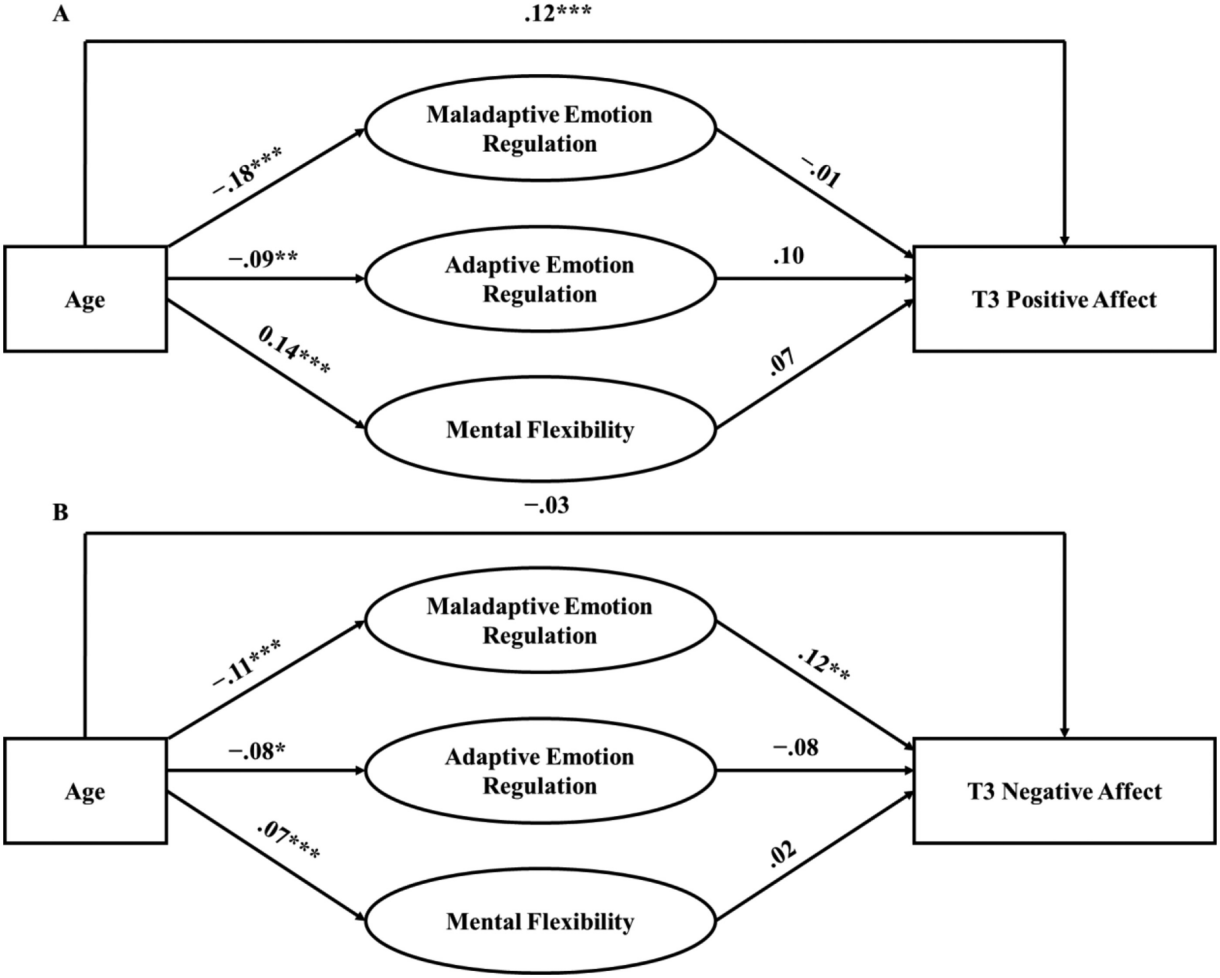
The Relationship Between Age, Use of Emotion Regulation Strategies, Mental Flexibility, and T3 Affect *Note*. The figure depicts two structural equation models examining the relationship between age, use of emotion regulation strategies, and mental flexibility, measured at T1, and positive (A) and negative (B) affect at T3. Age and positive/negative affect were modelled as continuous observed variables and emotion regulation strategies and mental flexibility were modelled as latent variables. The maladaptive emotion regulation latent variable comprises bespoke items indexing frequency of use of maladaptive strategies. The adaptive emotion regulation latent variable comprises bespoke items indexing frequency of use of adaptive strategies. The mental flexibility latent variable comprises items from the MFQ (Parsons et al., 2022). Positive affect reflects the average of positive emotions (i.e., content, happy, relieved, calm, appreciative) and negative affect reflects the average of negative emotions (i.e., anxious, angry, afraid, sad, worried, irritable, concerned, stressed, distressed, lonely, bored, hopeless, frustrated, disappointed) experienced in the previous 2 weeks because of the COVID-19 outbreak and resulting changes to daily life. Positive/negative affect at T1, gender, ethnicity, country and COVID-19 risk were controlled for but are not depicted in the models for simplicity. The standardized estimates of the associations between age with adaptive and maladaptive emotion regulation and mental flexibility differ across (A) and (B), because in (A), positive affect at T1 is included as a covariate on mediators and outcome, whereas in (B), negative affect at T1 is included as a covariate on mediators and outcome. The latent variables were allowed to covary. Paths include standardized βs. MFQ = Mental Flexibility Questionnaire. * *p* < .05. ** *p* < .01. *** *p* < .001.

Both models (controlling for affect at T1, gender, ethnicity, country, and COVID-19 risk; see Tables 4 and 5 in the online supplemental materials for results without covariates) testing the mediating effect of emotion regulation strategy use and mental flexibility on the association between age and T3 affect demonstrated acceptable fit to the data (positive affect: χ^2^ = 1,761.391, *p* < .001, CFI = .877, TLI = .847, RMSEA = .062, SRMR = .061; negative affect: χ^2^ = 1,768.251, *p* < .001, CFI = .882, TLI = .853, RMSEA = .062, SRMR = .063). Standardized estimates for direct effects are presented in Figure 1 and Table 3 in the online supplemental materials. Standardized indirect effects are presented in Table 3.[Fig fig1][Table tbl3]

**Figure 2 fig2:**
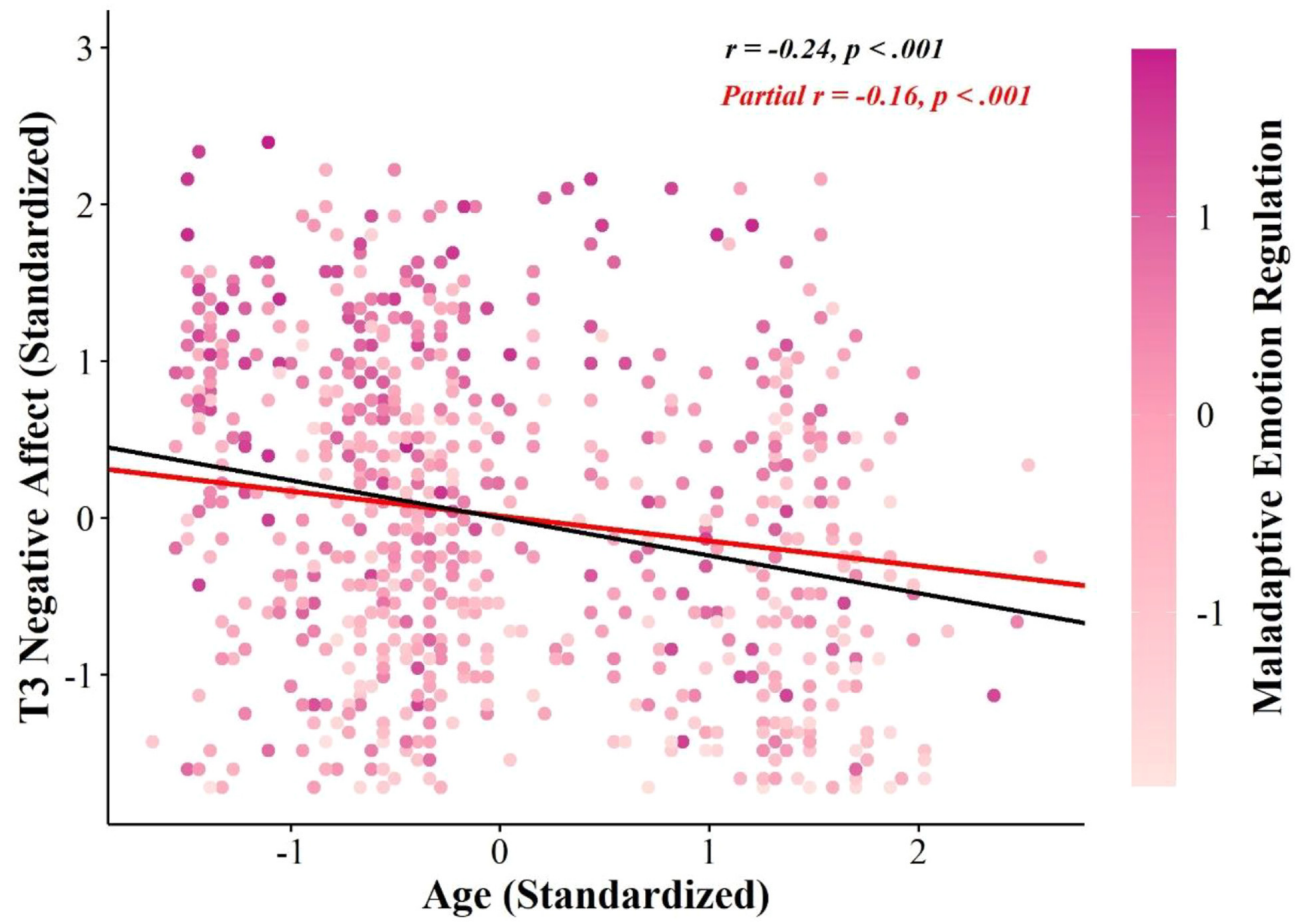
The Mediating Effect of Maladaptive Emotion Regulation on the Relationship Between Age and T3 Negative Affect *Note*. The figure shows the mediating effect of frequency of use of maladaptive emotion regulation strategies on the relationship between age and negative affect at our third assessment point. The correlation between age and T3 negative affect (shown in black) was calculated by regressing standardized T3 negative affect on standardized age. To calculate the partial correlation between age and T3 negative affect (shown in red), latent factor scores for the maladaptive emotion regulation latent variable were first extracted from our emotion regulation strategies measurement model. Standardized T3 negative affect was then regressed on standardized age, controlling for maladaptive emotion regulation factor scores. See the online article for the color version of this figure.

Use of maladaptive emotion regulation strategies partially accounted for variance in the relationship between age and negative affect at T3 (Figure 2). That is, younger age was associated with more frequent use of maladaptive emotion regulation strategies, which in turn, was associated with more negative affect at T3, over and above negative affect at T1. Use of maladaptive emotion regulation strategies did not significantly account for variance in the relationship between age and positive affect at T3, over and above positive affect at T1. Similarly, use of adaptive emotion regulation strategies and mental flexibility did not significantly account for variance in the relationship between age and positive or negative affect at T3, over and above affect at T1. Notably, younger age was associated with more frequent use of adaptive emotion regulation and lower mental flexibility; however, neither use of adaptive emotion regulation strategies or mental flexibility were significantly associated with positive or negative affect at T3. There were, however, significant cross-sectional associations between use of emotion regulation strategies and mental flexibility with affect at T1; such that more frequent use of maladaptive emotion regulation strategies was associated with less positive and more negative affect at T1; more frequent use of adaptive emotion regulation strategies was associated with more positive affect at T1; and greater mental flexibility was associated with more positive and less negative affect at T1. There were no significant indirect effects when T2 affect was included as the outcome variable (see Tables 6 and 7 in the online supplemental materials).[Fig fig2]

### The Relationship Between Age, Emotion Regulation Strategies, Mental Flexibility, Change in Affect, and Mental Health Problems

**Figure 3 fig3:**
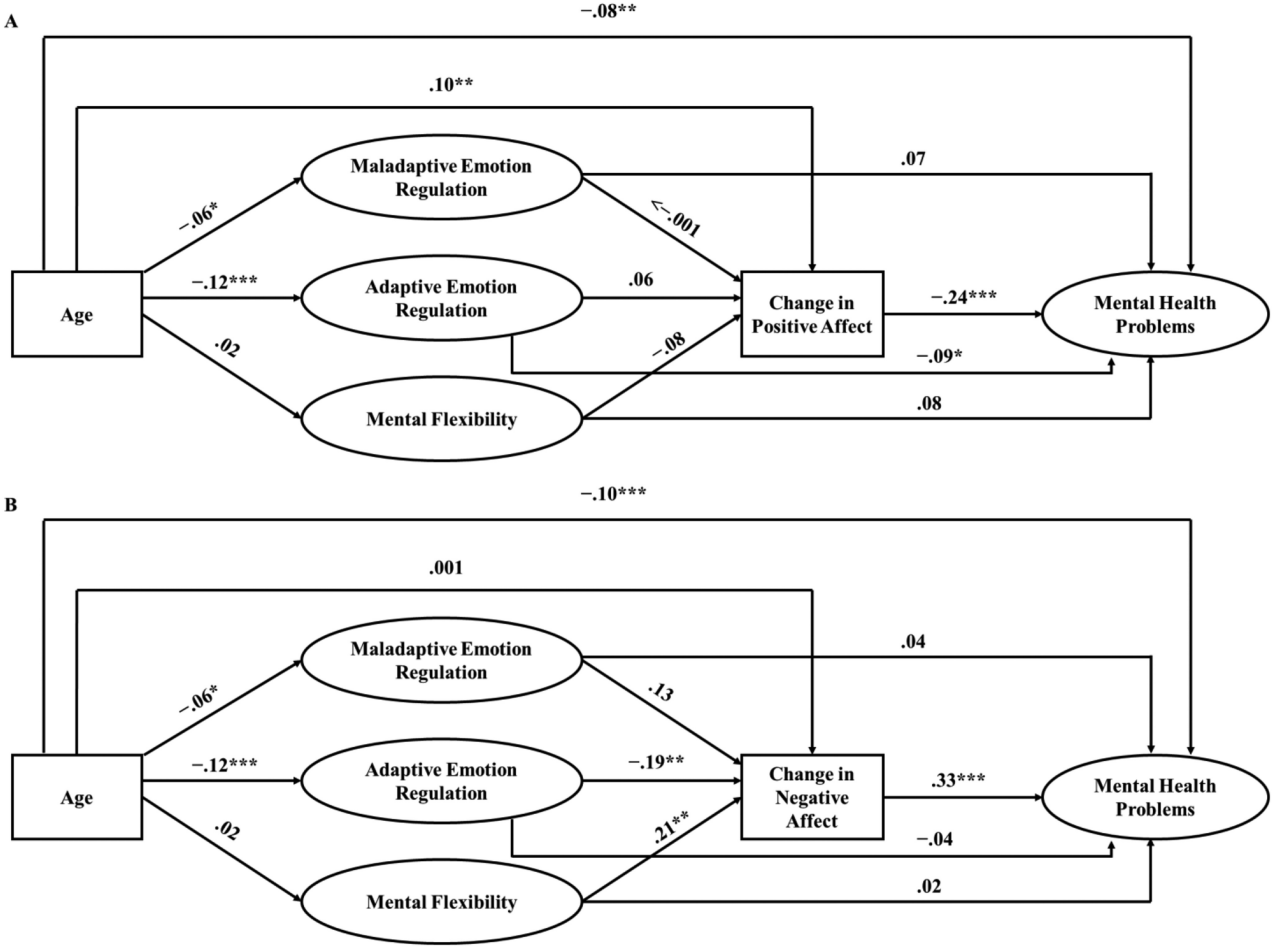
The Relationship Between Age, Use of Emotion Regulation Strategies, Mental Flexibility, Change in Affect, and Mental Health Problems *Note*. Figure 3 depicts two structural equation models examining the relationship between age, emotion regulation strategies, and mental flexibility measured at T1, change in positive (Figure 3A) and negative (Figure 3B) affect from T1 to T3, and mental health problems at T3. Age and positive/negative affect were modelled as continuous observed variables and emotion regulation and mental flexibility were modelled as latent variables. The maladaptive emotion regulation latent variable comprises bespoke items indexing frequency of use of maladaptive strategies. The adaptive emotion regulation latent variable comprises bespoke items indexing frequency of use of adaptive strategies. The mental flexibility latent variable comprises items from the MFQ (Parsons et al., 2022). Mental health problems were modelled as a higher order latent variable, as in Minihan et al. (2022), comprising depression symptoms measured with the PHQ-8 (Kroenke et al., 2001), anxiety symptoms measured with the GAD-7 (Spitzer et al., 2006), and mental wellbeing, measured with the seven-item WEMWBS (Stewart-Brown et al., 2009). Positive affect reflects the average of positive emotions (i.e., content, happy, relieved, calm, appreciative) and negative affect reflects the average of negative emotions (i.e., anxious, angry, afraid, sad, worried, irritable, concerned, stressed, distressed, lonely, bored, hopeless, frustrated, disappointed) experienced in the previous 2 weeks because of the COVID-19 outbreak and resulting changes to daily life. Mental health problems at T1, gender, ethnicity, country and COVID-19 risk were contolled for but are not depicted in the model for simplicity. The latent variables were allowed to covary. Paths include standardized βs. MFQ = Mental Flexibility Questionnaire, GAD-7 = seven-item General Anxiety Disorder scale, WEMWBS = Warwick-Edinburgh Mental Wellbeing Scale. * *p* < .05. ** *p* < .01. *** *p* < .001.

Both models (controlling for mental health problems at T1, gender, ethnicity, country, and COVID-19 risk; see Tables 9 and 10 in the online supplemental materials for results without covariates) testing the serial mediating effect of emotion regulation strategy use and mental flexibility, and in turn change in positive/negative affect from T1 to T3, on the association between age and T3 mental health problems demonstrated acceptable fit to the data (positive affect: χ^2^ = 10,162.232, *p* < .001, CFI = .866, TLI = .858, RMSEA = .043, SRMR = .065; negative affect: χ^2^ = 10,161.698, *p* < .001, CFI = .866, TLI = .859, RMSEA = .043, SRMR = .065). Standardized estimates for direct effects are presented in Figure 3 and Table 8 in the online supplemental materials. Standardized indirect effects are presented in Table 4.[Fig fig3][Table tbl4]

More frequent use of adaptive emotion regulation strategies and, in turn, change in negative affect from T1 to T3, partially accounted for variance in the relationship between age and mental health problems at T3, over and above mental health problems at T1. That is, younger age was associated with more frequent use of adaptive emotion regulation strategies, which in turn, was associated with a smaller increase (or greater decrease) in negative affect across time. Change in negative affect, in turn, was associated with less mental health problems at T3. Conversely, there was no significant serial mediating effect of use of maladaptive emotion regulation strategies or mental flexibility, and in turn, changes in negative or positive affect from T1 to T3, on the relationship between age and T3 mental health problems. There were, however, significant cross-sectional associations between emotion regulation strategies and mental flexibility with mental health problems at T1; more frequent use of maladaptive emotion regulation strategies was significantly associated with more mental health problems at T1, whereas more frequent use of adaptive emotion regulation strategies and mental flexibility were significantly associated with less mental health problems at T1. There were no significant indirect effects when change in affect from T1 to T2 was included as the mediator and T2 mental health problems as the outcome variable (see Tables 11 and 12 in the online supplemental materials).

## Discussion

The COVID-19 pandemic profoundly changed the way we live our lives, taking a considerable toll on the mental health and well-being of individuals across the globe. Converging evidence indicates that the mental health of younger people, in particular, has deteriorated during this pandemic (Racine et al., 2021). Given the transdiagnostic role emotion regulation strategies play in mental health problems and the developing nature of emotion regulation across adolescence (Ahmed et al., 2015; Silvers, 2022), here we examined the role of use of emotion regulation strategies and mental flexibility in affect and mental health problems across the lifespan during a year of the COVID-19 pandemic.

In line with recent research (Carstensen et al., 2020; Cunningham et al., 2021; Klaiber et al., 2021; Young et al., 2021), we found that younger relative to older individuals experienced less positive affect and more negative affect across the first year of the pandemic. More frequent use of maladaptive emotion regulation strategies partially accounted for variance in the relationship between age and negative affect at our third assessment point. Conversely, use of adaptive emotion regulation strategies and mental flexibility did not significantly explain any age-related variance in average positive or negative affect at T3. In a serial mediation model, we found that more frequent use of adaptive emotion regulation and, in turn, *change* in negative affect from T1 to T3, partially accounted for variance in the relationship between age and mental health problems at our third assessment point. Conversely, there was no significant serial mediating effect of use of maladaptive emotion regulation strategies or mental flexibility, and changes in negative or positive affect on the relationship between age and mental health problems at our third assessment point. Together, these findings demonstrate that more frequent use of maladaptive emotion regulation strategies may have contributed to the relatively heightened level of negative affectivity observed in younger people throughout the pandemic. At the same time, more frequent use of adaptive emotion regulation strategies in younger people may have buffered against an *increase* in (or helped decrease) negative affect across the course of the pandemic, in turn reducing mental health problems. Reducing the use of maladaptive emotion regulation strategies and augmenting the use of adaptive emotion regulation strategies amongst younger people, especially in the context of a novel, prolonged, and uncontrollable stressor (i.e., the COVID-19 pandemic), may thus be a promising means of improving negative affect amongst younger people and, in turn, mental health.

Indeed, younger people experienced on average more negative and less positive affect across the first year of the pandemic, with increased negative and reduced positive affect being hallmark symptoms of emotional disorders (Joormann & Vanderlind, 2014). Such age-related variability in affect is not unique to the pandemic, with developmental studies showing that adolescence is characterized by a shift in daily affective experiences, including increased negative affectivity as well as heightened intensity and variability of emotional states (McLaughlin et al., 2015). The experience of more novel, frequent, and intense emotions, in turn, heightens emotion regulation demands (Schäfer et al., 2017). Thus, the observed relationships between younger age and more frequent use of maladaptive and adaptive emotion regulation strategies may have been driven, in part, by the increased regulation demands experienced by younger people.

The stressors experienced during the pandemic differed greatly between younger and older individuals. These varying stressors may in turn have been associated with different emotion regulation demands. For example, younger people experienced substantial and prolonged disruptions to their education, with a study of 1,054 Canadian adolescents aged 14–18 years finding that 72% of adolescents were very worried that the pandemic would impact their school year (Ellis et al., 2020). In contrast, while many working adults were required to work from home during the pandemic, research found that remote work benefitted productivity and reduced stress in this age group (e.g., George et al., 2022). The intensity of the experienced stressors, work in adults showed, was also associated with the type of emotion regulation strategies that were selected (Blanke et al., 2022). Specifically, reappraisal and acceptance were used less, and rumination was used more, when stressors were more intense. Stressor type and intensity, then, may have varied across age groups and partially determined emotion regulation strategy selection.

Individuals across the lifespan have differing emotional goals, which may have additionally contributed to the observed age-related differences in reported strategy use and affect. For example, amongst individuals aged 14–86 years, adolescents more frequently reported contra-hedonic motivations of wanting to maintain or enhance negative affect or reduce positive affect, whereas older adults more frequently reported pro-hedonic motivations to maintain positive affect or dampen negative affect (Riediger et al., 2009). Future research examining age-related differences in strategy use and effectiveness, current stressors and stressor intensity, and emotional goals using, for example, an experience sampling approach, will help tease apart these effects.

While research has shown that use of maladaptive emotion regulation strategies is associated with increased negative affect in the short and long term (Aldao et al., 2010; Boemo et al., 2022; Gross & John, 2003; Joormann & Vanderlind, 2014; Moberly & Watkins, 2008; Nezlek & Kuppens, 2008), we found that more frequent use of maladaptive emotion regulation strategies was associated with average levels of negative affect, as opposed to an increase in negative affect across time. More frequent use of maladaptive emotion regulation strategies was also not significantly prospectively associated with mental health problems. It is possible that more frequent use of maladaptive emotion regulation strategies served to maintain already heightened levels of negative affect. Alternatively, the three items we used to assess use of maladaptive emotion regulation strategies may not have been sensitive enough to detect changes in negative affect across time or prospective associations with mental health problems. Indeed, there was a large cross-sectional association between more frequent use of maladaptive emotion regulation strategies and more mental health problems at T1, in line with meta-analytic findings showing medium to large effect sizes for the association between mental health problems and rumination, suppression, and avoidance (Aldao et al., 2010). Alternatively, the relationship between maladaptive emotion regulation with negative affect and mental health problems may be bidirectional, whereby increased negative emotions and mental health problems may lead to more frequent use of maladaptive emotion regulation strategies. A longitudinal study conducted during the COVID-19 pandemic supports this conclusion showing bidirectional associations between suppression and symptoms of depression and anxiety (Dawel et al., 2021). The present study assessed the use of emotion regulation strategies only at the first assessment, and consequently potential bidirectional associations between emotion regulation, affect, and mental health could not be examined, as well as potential changes in the use of emotion regulation strategies across time. Regardless of the nature of the relationship, the fact that more frequent use of maladaptive emotion regulation strategies partially accounted for variance in the relationship between younger age and increased negative affect at T3 (over and above negative affect at T1) suggests that reducing maladaptive emotion regulation in young people may be a promising means of reducing negative affectivity in young people.

In contrast to previous research showing increased use of adaptive emotion regulation strategies from adolescence to adulthood (Zimmermann & Iwanski, 2014), we found that younger age was associated with a greater tendency to use adaptive emotion regulation strategies. A greater tendency to use adaptive emotion regulation strategies in turn was associated with less increase (or a greater decrease) in negative affect across time, which mediated the association between age and T3 mental health problems. This finding needs to be interpreted, however, in the context of overall higher negative affect in young people at each assessment time point. It suggests that those young people who were able to deploy adaptive emotion regulation strategies did so successfully, benefiting their affective states and mental health. This is in line with work showing that, following a negative mood induction, early adolescents were able to successfully implement distraction, reappraisal, and acceptance to down-regulate negative affect (Wante et al., 2018). In contrast to the study by Wante et al. (2018), however, which found that adaptive emotion regulation strategies also increased positive affect in early adolescents; in the present study, more frequent use of adaptive emotion regulation strategies was not prospectively associated with positive affect during the pandemic. It is possible that the items we used to assess emotion regulation tapped more into the down-regulation of negative affect as opposed to the up-regulation of positive affect. Indeed, despite the more frequent use of adaptive emotion regulation strategies in younger people, younger people reported less positive affect and more negative affect, on average, across the first year of the pandemic. Thus, encouraging the use of emotion regulation strategies that also serve to up-regulate positive affect (e.g., savoring) in young people may be another promising intervention target.

In the present study, we examined emotion regulation beyond the frequency of use of maladaptive and adaptive strategies, to include a measure of mental flexibility as a cognitive correlate of emotion regulation flexibility. Greater mental flexibility was associated with more positive and less negative affect at T1, as well as less T1 mental health problems. However, it was not significantly prospectively associated with positive affect or mental health problems. Surprisingly, greater mental flexibility was associated with a greater increase (or less decrease) in negative affect across the first year of the pandemic. Given the wealth of evidence linking mental flexibility with better psychological functioning (Kashdan & Rottenberg, 2010), and that greater mental flexibility was strongly associated with less negative affect at T1, it is possible that individuals with higher mental flexibility may simply have had less negative affect to decrease in the first place.

The indirect effects from age to negative affect via maladaptive emotion regulation, and from age to mental health problems via adaptive emotion regulation and change in negative affect, only emerged after 6 months, but not at the 3-month assessment. While speculative, it is possible that more frequent use of emotion regulation strategies may drive sustained changes in affect over the longer as opposed to the shorter term. The lack of consistency in findings across timepoints, however, suggests that the results should be interpreted with caution and that replication of findings will be important.

The findings of the current study should be considered in light of a number of limitations. First, the CORAL study collected a convenience, nonprobability sample, and the findings, therefore, may not be representative at a population level (Pierce et al., 2020). Indeed, the majority of our sample identified as female, White, and of high socioeconomic status. This limits the generalizability of our findings across other samples. The lack of prepandemic data also limits the conclusions we can draw with regard to the role emotion regulation strategies and mental flexibility have played in mental health across the lifespan beyond the COVID-19 pandemic. Moreover, while we attempted to capture individual differences in the capacity to flexibly deploy situationally appropriate emotion regulation strategies by including a measure of mental flexibility, this measure assessed mental flexibility beyond emotion regulation flexibility. Future research should therefore extend these findings to include a measure that specifically assesses emotion regulation flexibility.

Despite these limitations, the current findings offer important insights into the age-related differences in affect and mental health that are increasingly being reported in studies conducted during the COVID-19 pandemic. Our findings suggest that more frequent use of maladaptive emotion regulation strategies may have contributed to the increased negative affectivity observed in younger people. While more frequent use of adaptive emotion regulation strategies in younger people was associated with less negative affect, and in turn, less mental health problems, younger people experienced on average more negative and less positive affect across the first year of the pandemic. Reducing habitual use of maladaptive emotion regulation strategies in young people, and augmenting strategies that up-regulate positive affect, may therefore be promising intervention targets.

## Supplementary Material

10.1037/emo0001238.supp

## Figures and Tables

**Table 1 tbl1:** Summary of Participant Characteristics

Participant characteristics	*n* (%)
Age	
11–24 years old	436 (18.42%)
25–64 years old	1,555 (65.69%)
65–100 years old	351 (14.83%)
Missing	25 (1.06%)
Gender identity	
Female	2,129 (89.95%)
Male	211 (8.91%)
Other	21 (0.89%)
Prefer not to say	6 (0.25%)
Country	
United Kingdom	1,075 (45.42%)
United States	699 (29.53%)
Australia	593 (25.05%)
Ethnicity	
White	1,995 (84.32%)
Asian	105 (4.44%)
Mixed	71 (3%)
Hispanic	44 (1.86%)
Black	16 (0.68%)
Aboriginal or Torres Strait Islander	10 (0.42%)
Other	95 (4.02%)
Prefer not to say	30 (1.27%)
Missing	1 (0.04%)
SES	
High	1,635 (69.07%)
Middle	654 (27.63%)
Low	5 (0.21%)
Missing	73 (3.09%)
*Note*. SES = socioeconomic status. For participants over the age of 18 years, SES was operationalized as their highest educational attainment. For participants under the age of 18 years, SES was operationalized as the average of their parent’s highest educational attainment; high = university, middle = high school or professional/vocational training, low = primary school.	

**Table 2 tbl2:** Association Between Age and Affect

Coefficient	Positive affect				Negative affect			
	*b*	*SE*	95% CI	*p*	*b*	*SE*	95% CI	*p*
(Intercept)	3.250	0.084	[3.085–3.415]	<.001	2.863	0.092	[2.682–3.043]	<.001
Wave	−0.018	0.016	[−0.050 to 0.013]	.256	**−0.120**	**0.014**	**[−0.148 to −0.092]**	<.001
Age	**0.008**	**0.001**	**[0.006–0.010]**	<.001	**−0.015**	**0.001**	**[−0.017 to −0.012]**	<.001
Female	−0.021	0.065	[−0.148 to 0.106]	.744	**0.169**	**0.071**	**[0.030–0.308]**	.017
White	**−0.176**	**0.057**	**[−0.288 to −0.064]**	.002	**−0.140**	**0.062**	**[−0.262 to −0.018]**	.024
United Kingdom	**−0.256**	**0.050**	**[−0.354 to −0.159]**	<.001	**0.614**	**0.054**	**[0.507–0.720]**	<.001
United States	**−0.187**	**0.053**	**[−0.292 to −0.083]**	<.001	**0.634**	**0.058**	**[0.520–0.749]**	<.001
COVID-19 risk	−0.012	0.013	[−0.037 to 0.013]	.360	**0.042**	**0.014**	**[0.015–0.069]**	.003
Wave × Age	0.002	0.001	[<0.001 to 0.003]	.050	0.001	0.001	[<−0.001 to 0.003]	.087
Random effects								
σ^2^	.51				.38			
τ_00_	.54_id_				.79_id_			
ICC	.52				.68			
*N*	2,305_id_				2,306_id_			
Observations	3,951				3,954			
Marginal *R*^2^/conditional *R*^2^	.036/.533				.126/.719			
*Note*. Positive and negative affect were assessed with a series of bespoke items and reflect the average of positive (i.e., content, happy, relieved, calm, appreciative) and negative (i.e., anxious, angry, afraid, sad, worried, irritable, concerned, stressed, distressed, lonely, bored, hopeless, frustrated, disappointed) emotions experienced in the previous 2 weeks because of the COVID-19 outbreak and resulting changes to daily life. Time indexes the three assessment timepoints, modelled as a continuous variable, with the first timepoint coded as 0. Age was measured in years and mean centered. For gender, “other” is the reference group, which includes responses options “male” and “other.” For ethnicity, “other” is the reference group, which includes responses options: “Asian,” “Hispanic,” “Black,” “Aboriginal or Torres Strait Islander,” “Mixed,” or “other.” For country, Australia is the reference group. COVID-19 risk was measured with a series of bespoke items indexing quarantining, diagnosis, hospitalization and death, described further in the online supplemental material 1. Bold text denotes statistical significance. CI = confidence interval; ICC = intraclass correlation coefficient.								

**Table 3 tbl3:** Indirect Effects in the Relationship Between Age, Use of Emotion Regulation Strategies, Mental Flexibility, and T3 Affect

Indirect effects	Positive affect				Negative affect			
	β	*SE*	95% CI	*p*	β	*SE*	95% CI	*p*
Age → maladaptive emotion regulation → T3 affect	.001	.009	[−.016 to .018]	.918	**−.013**	**.006**	**[−.024** to **−.002]**	**.020**
Age → adaptive emotion regulation → T3 affect	−.009	.006	[−.020 to .003]	.128	.006	.004	[−.002 to .014]	.127
Age → mental flexibility → T3 affect	.010	.008	[−.005 to .026]	.187	.001	.003	[−.005 to .008]	.722
*Note*. Age and positive/negative affect were modelled as continuous observed variables and emotion regulation strategies and mental flexibility were modelled as latent variables. The maladaptive emotion regulation latent variable comprises bespoke items indexing frequency of use of maladaptive strategies. The adaptive emotion regulation latent variable comprises bespoke items indexing frequency of use of adaptive strategies. The mental flexibility latent variable comprises items from the MFQ (Parsons et al., 2022). Positive affect reflects the average of positive emotions (i.e., content, happy, relieved, calm, appreciative) and negative affect reflects the average of negative emotions (i.e., anxious, angry, afraid, sad, worried, irritable, concerned, stressed, distressed, lonely, bored, hopeless, frustrated, disappointed) experienced in the previous 2 weeks because of the COVID-19 outbreak and resulting changes to daily life. T1 affect, gender, ethnicity, country, and COVID-19 risk were included as covariates. Paths include standardized βs, standardized *SE*s, and standardized 95% confidence intervals. CI = confidence interval; MFQ = Mental Flexibility Questionnaire. Bold text denotes statistical significance.								

**Table 4 tbl4:** Indirect Effects in the Relationship Between Age, Use of Emotion Regulation Strategies, Mental Flexibility, Change in Affect from T1 to T3, and T3 Mental Health Problems

Indirect effects	Positive affect				Negative affect			
	β	*SE*	95% CI	*p*	β	*SE*	95% CI	*p*
Age → maladaptive emotion regulation → change in affect → T3 mental health problems	<.001	.001	[−.002 to .002]	.997	−.003	.002	[−.006 to .001]	.177
Age → adaptive emotion regulation → change in affect → T3 mental health problems	.002	.002	[−.002 to .005]	.385	**.007**	**.003**	**[.001–.014]**	**.023**
Age → mental flexibility → change in affect → T3 mental health problems	<.001	<.001	[−.001 to .001]	.459	.001	.001	[−.001 to .004]	.343
Age → maladaptive emotion regulation → T3 mental health problems	−.004	.003	[−.011 to .002]	.194	−.002	.003	[−.008 to .003]	.398
Age → adaptive emotion regulation → T3 mental health problems	.011	.005	[<.001 to .021]	.043	.005	.004	[−.003 to .012]	.264
Age → mental flexibility → T3 mental health problems	.001	.002	[−.002 to .004]	.398	<.001	.001	[−.001 to .002]	.720
Age → change in affect → T3 mental health problems	**−.024**	**.009**	**[−.042** to **−.006]**	**.008**	<.001	.010	[−.020 to .021]	.968
*Note*. Age and positive/negative affect were modelled as continuous observed variables and emotion regulatory processes were modelled as latent variables. The maladaptive emotion regulation latent variable comprises bespoke items indexing frequency of use of maladaptive strategies. The adaptive emotion regulation latent variable comprises bespoke items indexing frequency of use of adaptive strategies. The mental flexibility latent variable comprises items from the MFQ (Parsons et al., 2022). Mental health problems were modelled as a higher order latent variable, as in Minihan et al. (2022), comprising depression symptoms measured with the PHQ-8 (Kroenke et al., 2001), anxiety symptoms measured with the GAD-7 (Spitzer et al., 2006), and mental wellbeing, measured with the seven-item WEMWBS (Stewart-Brown et al., 2009). Positive affect reflects the average of positive emotions (i.e., content, happy, relieved, calm, appreciative) and negative affect reflects the average of negative emotions (i.e., anxious, angry, afraid, sad, worried, irritable, concerned, stressed, distressed, lonely, bored, hopeless, frustrated, disappointed) experienced in the previous 2 weeks because of the COVID-19 outbreak and resulting changes to daily life. T1 mental health problems, gender, ethnicity, country, and COVID-19 risk were included as covariates. Paths included standardized βs, standardized *SE*s, and standardized 95% confidence intervals. CI = confidence interval; MFQ = Mental Flexibility Questionnaire; PHQ-8 = eight-item Patient Health Questionnaire; GAD-7 = seven-item General Anxiety Disorder scale, WEMWBS = Warwick-Edinburgh Mental Wellbeing Scale. Bold text denotes statistical significance.
