# Supplementary material for: Affect and Mental Health Across the Lifespan During a Year of the COVID-19 Pandemic: The Role of Emotion Regulation Strategies and Mental Flexibility
Source: Emotion. 2023 May 18;24(1):67–80. doi: 10.1037/emo0001238 (PMC11064816; doi:10.1037/emo0001238)
Supplement: Supplementary file 1 [file EMO-2022-3336_Supplementary_Materials.docx]

**Affect and Mental Health Across the Lifespan During a Year of the COVID-19 Pandemic: The Role of Emotion Regulation Strategies and Mental Flexibility**

**Supplementary Materials**

**SM 1**

***COVID–19 Risk***

A number of bespoke items were included in the study to assess COVID–19–related risk. First, participants indicated whether they had ever been tested for COVID–19. Response options included: Yes, I was tested for COVID–19 and am waiting for the results; Yes, I was tested for COVID–19 and the results were positive; Yes, I was tested for COVID–19 and the results were negative; Yes, I was tested for COVID–19 but I do not want to share the results; No, I was not tested for COVID–19, but I was given a medical diagnosis of COVID–19 (they assume I have COVID–19); No, I was not tested for COVID–19, because I could not get a test; and No, I have not tried to get a test. Next, participants responded to a number of binary response options, indicating whether they or anyone in their house had ever been quarantined due to possibly having COVID–19; whether they had been hospitalized due to COVID–19; or whether they knew anyone personally who had been diagnosed with, hospitalized due to, or passed away from COVID–19. Those participants who indicated they knew someone who had been hospitalized with or passed away from COVID–19 were additionally asked about the nature of their relationship with these people (i.e., parent, child, sibling, grandparent, friend, other relative, other non–relative).

The COVID–19 risk items were weighted as follows: 0.5 for quarantining for reasons other than diagnosis; 0.5 for knowing others diagnosed with COVID–19; 1 for quarantining due to diagnosis; 1.5 for hospitalization of other relative or other non–relative; 2 for death of other relative or other non–relative; 3 for hospitalization of self, parent, child, sibling, grandparent, or friend; and 4 for death of parent, child, sibling, grandparent, or friend. A composite COVID–19 risk score was computed by adding the highest value from the variables assessing diagnosis in other person, quarantining due to diagnosis or other reason, hospitalization of other relative or other non–relative, and death of other relative or other non–relative, to the variables assessing hospitalization of self/kin/friend and death of kin/friend. While these variables were assessed at all timepoints, only T1 COVID–19 risk was controlled for in analyses, allowing us to maintain our T1 sample size. This variable was included as a covariate in order to control for the potential impact of COVID-19-related exposure and health risk on mental health problems (Hossain et al., 2020).

**SM Table 1**

*Descriptives and Correlations Between Study Variables*

**

*Note.* Depression symptoms were measured with the 8-item Patient Health Questionnaire (PHQ-8; Kroenke et al., 2001). Anxiety symptoms were measured with the 7-item General Anxiety Disorder scale (GAD-7; Spitzer et al., 2006). Mental wellbeing was measured with the with the 7-item Warwick-Edinburgh Mental Wellbeing Scale (WEMWBS; Stewart-Brown et al., 2009; this measure was reverse-coded in subsequent analyses, but non-reverse-coded scores are shown here for ease of interpretation). Positive and negative affect were assessed with a series of bespoke items and reflect the average of positive (i.e., content, happy, relieved, calm, appreciative) and negative (i.e., anxious, angry, afraid, sad, worried, irritable, concerned, stressed, distressed, lonely, bored, hopeless, frustrated, disappointed) emotions experienced in the previous two weeks because of the COVID-19 outbreak and resulting changes to daily life. A series of bespoke items assessed frequency of use of adaptive and maladaptive emotion regulation strategies. Mental flexibility was assessed with the Mental Flexibility Questionnaire (MFQ; Parsons et al., 2022). COVID-19 risk was measured with a series of bespoke items indexing quarantining, diagnosis, hospitalization and death, described further in SM1. **p* < .05, ** *p* < .01, *** *p* < .001.

**SM Table 2**

Association Between Age and Affect (Without Covariates)

|  | **Positive Affect** | | | | **Negative Affect** | | | |
| --- | --- | --- | --- | --- | --- | --- | --- | --- |
| *Coefficient* | *b* | *SE* | *95% CI* | *p* | *b* | *SE* | *95% CI* | *p* |
| (Intercept) | 2.911 | 0.021 | 2.870 – 2.953 | **<0.001** | 3.369 | 0.023 | 3.324 – 3.414 | **<0.001** |
| Wave | −0.020 | 0.016 | −0.051 – 0.011 | 0.212 | **−0.119** | **0.014** | **−0.147 – −0.092** | **<0.001** |
| Age | **0.008** | **0.001** | **0.005 – 0.010** | **<0.001** | **−0.016** | **0.001** | **−0.018 – −0.013** | **<0.001** |
| Wave * Age | 0.002 | 0.001 | <−0.001 – 0.003 | 0.070 | 0.001 | 0.001 | <−0.001 – 0.003 | 0.094 |
| **Random Effects** | | | | | | | | |
| σ^2^ | .51 | | | | .37 | | | |
| τ_00_ | .56 _id_ | | | | .89 _id_ | | | |
| ICC | .52 | | | | .70 | | | |
| N | 2341 _id_ | | | | 2342 _id_ | | | |
| Observations | 4005 | | | | 4008 | | | |
| Marginal R^2^ / Conditional R^2^ | .022 / .535 | | | | .060 / .722 | | | |

*Note.* Positive and negative affect were assessed with a series of bespoke items and reflect the average of positive (i.e., content, happy, relieved, calm, appreciative) and negative (i.e., anxious, angry, afraid, sad, worried, irritable, concerned, stressed, distressed, lonely, bored, hopeless, frustrated, disappointed) emotions experienced in the previous two weeks because of the COVID-19 outbreak and resulting changes to daily life. Time indexes the three assessment timepoints, modelled as a continuous variable, with the first timepoint coded as 0. Age was measured in years and mean centered.

**SM Table 3**

*Direct Effects in the Relationship Between Age, Emotion Regulation Strategies, Mental Flexibility, and T3 Affect (With Covariates)*

|  | **Positive Affect** | | | | **Negative Affect** | | | |
| --- | --- | --- | --- | --- | --- | --- | --- | --- |
|  | ***β*** | ***SE*** | ***95% CI*** | ***p*** | ***β*** | ***SE*** | ***95% CI*** | ***p*** |
| Maladaptive emotion regulation |  |  |  |  |  |  |  |  |
| ~Age | **−.177** | **.024** | **−.224 – −.129** | **<.001** | **−.107** | **.024** | **−.155 – −.059** | **<.001** |
| ~Female | −.005 | .023 | −.051 – .041 | .828 | −.032 | .022 | −.075 – .011 | .150 |
| ~White | **−.072** | **.025** | **−.121 – −.022** | **.005** | −.043 | .023 | −.089 – .003 | .064 |
| ~UK | .007 | .030 | −.053 – .066 | .823 | **−.071** | **.029** | **−.129 – −.013** | **.016** |
| ~US | .030 | .029 | −.028 – .088 | .310 | −.048 | .029 | −.105 – .009 | .097 |
| ~COVID-19 Risk | .003 | .024 | −.044 – .050 | .889 | −.024 | .023 | −.068 – .021 | .295 |
| ~T1 Affect | **−.233** | **.026** | **−.284 – −.181** | **<.001** | **.460** | **.026** | **.409 – .511** | **<.001** |
| Adaptive Emotion Regulation |  |  |  |  |  |  |  |  |
| ~Age | **−.091** | **.032** | **−.153 – −.030** | **.004** | **−.078** | **.032** | **−.140 – −.016** | **.014** |
| ~Female | **.068** | **.029** | **.011 – .124** | **.019** | **.076** | **.029** | **.019 – .132** | **.009** |
| ~White | −.012 | .028 | −.067 – .043 | .673 | −.027 | .028 | −.083 – .029 | .344 |
| ~UK | **−.177** | **.032** | **−.239 – −.115** | **<.001** | **−.189** | **.032** | **−.253 – −.126** | **<.001** |
| ~US | −.038 | .031 | −.100 – .023 | .224 | −.047 | .032 | −.1091 – .016 | .143 |
| ~COVID–19 Risk | .054 | .026 | .004 – .104 | .035 | .056 | .026 | .005 – .107 | .032 |
| ~T1 Affect | **.204** | **.029** | **.148 – .261** | **<.001** | −.056 | .034 | −.122 – .010 | .099 |
| Mental Flexibility |  |  |  |  |  |  |  |  |
| ~Age | **.139** | **.019** | **.101 – .177** | **<.001** | **.070** | **.018** | **.034 – .106** | **<.001** |
| ~Female | −.022 | .021 | −.063 – .018 | .280 | .014 | .018 | −.022 – .050 | .446 |
| ~White | .004 | .021 | −.036 – .044 | .849 | **−.048** | **.021** | **−.088 – −.007** | **.022** |
| ~UK | **−.108** | **.024** | **−.155 – −.061** | **<.001** | −.025 | .023 | −.069 – .019 | .270 |
| ~US | −.016 | .024 | −.063 – .031 | .507 | **.069** | **.023** | **.024 – .114** | **.003** |
| ~COVID-19 Risk | −.018 | .020 | −.058 – .021 | .368 | .017 | .019 | −.020 – .055 | .360 |
| ~T1 Affect | **.476** | **.018** | **.441 – .511** | **<.001** | **−.605** | **.017** | **−.637 – −.572** | **<.001** |
| T3 Affect |  |  |  |  |  |  |  |  |
| ~Maladaptive Emotion Regulation | −.005 | .050 | −.102 – .092 | .918 | **.124** | **.045** | **.036 – .212** | **.006** |
| ~Adaptive Emotion Regulation | .096 | .056 | −.013 – .205 | .084 | −.080 | .042 | −.162 – .001 | .054 |
| ~Mental Flexibility | .074 | .055 | −.034 – .181 | .178 | .017 | .048 | −.077 – .112 | .721 |
| ~Age | **.118** | **.031** | **.058 – .178** | **<.001** | −.027 | .023 | −.072 – .018 | .245 |
| ~Female | −.021 | .032 | −.084 – .042 | .518 | .025 | .023 | −.020 – .070 | .272 |
| ~White | −.056 | .031 | −.117 – .005 | .072 | .003 | .025 | −.046 – .052 | .907 |
| ~UK | **−.134** | **.042** | **−.217 – −.051** | **.002** | **.287** | **.032** | **.222 – .353** | **<.001** |
| ~US | −.062 | .039 | −.138 – .015 | .115 | **.300** | **.031** | **.240 – .360** | **<.001** |
| ~COVID-19 Risk | .009 | .028 | −.046 – .063 | .758 | −.019 | .025 | −.067 – .029 | .446 |
| ~T1 Affect | **.438** | **.035** | **.370 – .506** | **<.001** | **.586** | **.037** | **.512 – .659** | **<.001** |

*Note.* Age and positive/negative affect were modelled as continuous observed variables and emotion regulation strategies and mental flexibility were modelled as latent variables. The maladaptive emotion regulation latent variable comprises bespoke items indexing frequency of use of maladaptive strategies. The adapative emotion regulation latent variable comprises bespoke items indexing frequency of use of adaptive strategies. The mental flexibility latent variable comprises items from the MFQ (Parsons et al., 2022). Positive affect reflects the average of positive emotions (i.e., content, happy, relieved, calm, appreciative) and negative affect reflects the average of negative emotions (i.e., anxious, angry, afraid, sad, worried, irritable, concerned, stressed, distressed, lonely, bored, hopeless, frustrated, disappointed) experienced in the previous two weeks because of the COVID–19 outbreak and resulting changes to daily life. For gender, ‘other’ is the reference group, which includes responses options ‘male’ and ‘other’. For ethnicity, ‘other’ is the reference group, which includes responses options: ‘Asian’, ‘Hispanic’, ‘Black’, ‘Aboriginal or Torres Strait Islander’, ‘Mixed’, or ‘other’. For country, Australia is the reference group. COVID–19 risk was measured with a series of bespoke items indexing quarantining, diagnosis, hospitalization and death, described further in SM1. Paths included standardized *β*s, standardized SEs, and standardized 95% confidence intervals.

**SM Table 4**

*Direct Effects in the Relationship Between Age, Emotion Regulation Strategies, Mental Flexibility, and T3 Affect (Without Covariates)*

|  | **Positive Affect** | | | | **Negative Affect** | | | |
| --- | --- | --- | --- | --- | --- | --- | --- | --- |
|  | ***β*** | ***SE*** | ***95% CI*** | ***p*** | ***β*** | ***SE*** | ***95% CI*** | ***p*** |
| Maladaptive Emotion Regulation |  |  |  |  |  |  |  |  |
| ~Age | **−.193** | **.024** | **−.239 – −.146** | **<.001** | **−.121** | **.024** | **−.168 – −.074** | **<.001** |
| ~T1 Affect | **−.232** | **.026** | **−.282 – −.181** | **<.001** | **.442** | **.025** | **.392 – .492** | **<.001** |
| Adaptive Emotion Regulation |  |  |  |  |  |  |  |  |
| ~Age | **−.081** | **.032** | **−.143 – −.019** | **.010** | **−.073** | **.031** | **−.135 – −.011** | **.020** |
| ~T1 Affect | **.213** | **.028** | **.158 – .269** | **<.001** | **−.076** | **.033** | **−.141 – −.011** | **.023** |
| Mental Flexibility |  |  |  |  |  |  |  |  |
| ~Age | **.144** | **.019** | **.107 – .181** | **<.001** | **.067** | **.018** | **.031 – .102** | **<.001** |
| ~T1 Affect | **.478** | **.018** | **.444 – .513** | **<.001** | **−.596** | **.016** | **−.627 – −.565** | **<.001** |
| T3 Affect |  |  |  |  |  |  |  |  |
| ~Maladaptive Emotion Regulation | −.002 | .049 | −.098 – .094 | .970 | **.127** | **.047** | **.036 – .219** | **.006** |
| ~Adaptive Emotion Regulation | .108 | .055 | .001 – .215 | .048 | **−.114** | **.042** | **−.197 – −.032** | **.007** |
| ~Mental Flexibility | .081 | .055 | −.026 – .188 | .137 | .045 | .050 | −.052 – .142 | .363 |
| ~Age | **.105** | **.031** | **.045 – .165** | **.001** | −.026 | .024 | −.073 – .022 | .288 |
| ~T1 Affect | **.447** | **.035** | **.378 – .516** | **<.001** | **.660** | **.036** | **.590 – .730** | **<.001** |

*Note.* Age and positive/negative affect were modelled as continuous observed variables and emotion regulation strategies and mental flexibility were modelled as latent variables. The maladaptive emotion regulation latent variable comprises bespoke items indexing frequency of use of maladaptive strategies. The adapative emotion regulation latent variable comprises bespoke items indexing frequency of use of adaptive strategies. The mental flexibility latent variable comprises items from the MFQ (Parsons et al., 2022). Positive affect reflects the average of positive emotions (i.e., content, happy, relieved, calm, appreciative) and negative affect reflects the average of negative emotions (i.e., anxious, angry, afraid, sad, worried, irritable, concerned, stressed, distressed, lonely, bored, hopeless, frustrated, disappointed) experienced in the previous two weeks because of the COVID–19 outbreak and resulting changes to daily life. Paths included standardized *β*s, standardized SEs, and standardized 95% confidence intervals.

**SM Table 5**

*Indirect Effects in the Relationship Between Age, Emotion Regulation Strategies, Mental Flexibility, and T3 Affect (Without Covariates)*

|  | **Positive Affect** | | | | **Negative Affect** | | | |
| --- | --- | --- | --- | --- | --- | --- | --- | --- |
|  | ***β*** | ***SE*** | ***95% CI*** | ***p*** | ***β*** | ***SE*** | ***95% CI*** | ***p*** |
| Age 🡪 Maladaptive Emotion Regulation 🡪 T3 Affect | <.001 | .009 | −.018 – .019 | .970 | **−.015** | **.006** | **−.028 – −.003** | **.017** |
| Age 🡪 Adaptive Emotion Regulation 🡪 T3 Affect | −.009 | .005 | −.019 – .002 | .106 | .008 | .005 | −.001 – .017 | .073 |
| Age 🡪 Mental Flexibility 🡪 T3 Affect | .012 | .008 | −.004 – .027 | .144 | .003 | .003 | −.004 – .010 | .379 |

*Note.* Age and positive/negative affect were modelled as continuous observed variables and emotion regulation strategies and mental flexibility were modelled as latent variables. The maladaptive emotion regulation latent variable comprises bespoke items indexing frequency of use of maladaptive strategies. The adapative emotion regulation latent variable comprises bespoke items indexing frequency of use of adaptive strategies. The mental flexibility latent variable comprises items from the MFQ (Parsons et al., 2022). Positive affect reflects the average of positive emotions (i.e., content, happy, relieved, calm, appreciative) and negative affect reflects the average of negative emotions (i.e., anxious, angry, afraid, sad, worried, irritable, concerned, stressed, distressed, lonely, bored, hopeless, frustrated, disappointed) experienced in the previous two weeks because of the COVID–19 outbreak and resulting changes to daily life. T1 affect was included as a covariate. Paths included standardized *β*s, standardized SEs, and standardized 95% confidence intervals.

**SM Table 6**

*Direct Effects in the Relationship Between Age, Emotion Regulation Strategies, Mental Flexibility, and T2 Affect (With Covariates)*

|  | **Positive Affect** | | | | **Negative Affect** | | | |
| --- | --- | --- | --- | --- | --- | --- | --- | --- |
|  | ***β*** | ***SE*** | ***95% CI*** | ***p*** | ***β*** | ***SE*** | ***95% CI*** | ***p*** |
| Maladaptive emotion regulation |  |  |  |  |  |  |  |  |
| ~Age | **−.177** | **.024** | **−.225 – −.129** | **<.001** | **−.107** | **.024** | **−.155 – −.060** | **<.001** |
| ~Female | −.005 | .023 | −.051 – .040 | .815 | −.031 | .022 | −.075 – .012 | .152 |
| ~White | **−.072** | **.025** | **−.121 – −.022** | **.005** | −.042 | .023 | −.088 – .003 | .069 |
| ~UK | .006 | .030 | −.053 – .066 | .833 | **−.072** | **.029** | **−.129 – −.014** | **.015** |
| ~US | .030 | .030 | −.028 – .088 | .315 | −.049 | .029 | −.105 – .008 | .093 |
| ~COVID-19 Risk | .004 | .024 | −.043 – .051 | .882 | −.024 | .022 | −.068 – .020 | .281 |
| ~T1 Affect | **−.233** | **.026** | **−.285 – −.182** | **<.001** | **.458** | **.027** | **.406 – .510** | **<.001** |
| Adaptive emotion regulation |  |  |  |  |  |  |  |  |
| ~Age | **−.092** | **.032** | **−.153 – −.030** | **.004** | **−.077** | **.032** | **−.139 – −.016** | **.014** |
| ~Female | **.068** | **.029** | **.011 – .124** | **.018** | **.076** | **.029** | **.019 – .132** | **.009** |
| ~White | −.012 | .028 | −.067 – .043 | .672 | −.027 | .028 | −.083 – .029 | .340 |
| ~UK | **−.177** | **.032** | **−.239 – −.115** | **<.001** | **−.189** | **.032** | **−.253 – −.125** | **<.001** |
| ~US | −.038 | .031 | −.100 – .023 | .223 | −.046 | .032 | −.108 – .017 | .150 |
| ~COVID-19 Risk | .055 | .026 | .004 – .105 | .033 | .056 | .026 | .005 – .107 | .033 |
| ~T1 Affect | **.205** | **.029** | **.148 – .262** | **<.001** | −.056 | .034 | −.122 – .010 | .096 |
| Mental Flexibility |  |  |  |  |  |  |  |  |
| ~Age | **.139** | **.019** | **.101 – .177** | **<.001** | **.070** | **.018** | **.034 – .106** | **<.001** |
| ~Female | −.022 | .021 | −.062 – .018 | .287 | .014 | .018 | −.022 – .050 | .444 |
| ~White | .004 | .021 | −.036 – .044 | .850 | **−.048** | **.021** | **−.088 – −.007** | **.022** |
| ~UK | **−.108** | **.024** | **−.155 – −.061** | **<.001** | −.025 | .023 | −.069 – .019 | .271 |
| ~US | −.016 | .024 | −.063 – .031 | .509 | **.069** | **.023** | **.025 – .114** | **.002** |
| ~COVID-19 Risk | −.018 | .020 | −.057 – .022 | .384 | .017 | .019 | −.020 – .055 | .363 |
| ~T1 Affect | **.476** | **.018** | **.441 – .511** | **<.001** | **−.605** | **.017** | **−.638 – −.572** | **<.001** |
| T2 Affect |  |  |  |  |  |  |  |  |
| ~Maladaptive emotion regulation | −.043 | .043 | −.128 – .042 | .319 | .070 | .040 | −.009 – .148 | .082 |
| ~Adaptive emotion regulation | .048 | .047 | −.045 – .141 | .309 | .006 | .035 | −.062 – .073 | .869 |
| ~Mental Flexibility | .063 | .052 | −.039 – .166 | .227 | −.084 | .043 | −.169 – .002 | .055 |
| ~Age | .056 | .027 | .003 – .110 | .039 | −.014 | .023 | −.059 – .031 | .540 |
| ~Female | −.058 | .026 | −.110 – −.006 | .028 | −.007 | .022 | −.050 – .036 | .754 |
| ~White | **−.078** | **.029** | **−.134 – −.021** | **.007** | −.020 | .025 | −.069 – .003 | .435 |
| ~UK | .004 | .037 | −.069 – .076 | **.**922 | .017 | .031 | −.045 – .078 | .594 |
| ~US | .024 | .035 | −.045 – .093 | .493 | .061 | .029 | .005 – .117 | .032 |
| ~COVID-19 Risk | .012 | .023 | −.032 – .056 | .595 | −.013 | .020 | −.052 – .027 | .528 |
| ~T1 Affect | **.505** | **.035** | **.437 – .572** | **<.001** | **.644** | **.034** | **.578 – .709** | **<.001** |

*Note.* Age and positive/negative affect were modelled as continuous observed variables and emotion regulation strategies and mental flexibility were modelled as latent variables. The maladaptive emotion regulation latent variable comprises bespoke items indexing frequency of use of maladaptive strategies. The adapative emotion regulation latent variable comprises bespoke items indexing frequency of use of adaptive strategies. The mental flexibility latent variable comprises items from the MFQ (Parsons et al., 2022). Positive affect reflects the average of positive emotions (i.e., content, happy, relieved, calm, appreciative) and negative affect reflects the average of negative emotions (i.e., anxious, angry, afraid, sad, worried, irritable, concerned, stressed, distressed, lonely, bored, hopeless, frustrated, disappointed) experienced in the previous two weeks because of the COVID–19 outbreak and resulting changes to daily life. For gender, ‘other’ is the reference group, which includes responses options ‘male’ and ‘other’. For ethnicity, ‘other’ is the reference group, which includes responses options: ‘Asian’, ‘Hispanic’, ‘African’, ‘Aboriginal or Torres Strait Islander’, ‘Mixed’, or ‘other’. For country, Australia is the reference group. COVID-19 risk was measured with a series of bespoke items indexing quarantining, diagnosis, hospitalization and death, described further in SM1. Paths included standardized *β*s, standardized SEs, and standardized 95% confidence intervals.

**SM Table 7**

*Indirect Effects in the Relationship Between Age, Emotion Regulation Strategies, Mental Flexibility, and T2 Affect (With Covariates)*

|  | **Positive Affect** | | | | **Negative Affect** | | | |
| --- | --- | --- | --- | --- | --- | --- | --- | --- |
|  | ***β*** | ***SE*** | ***95% CI*** | ***p*** | ***β*** | ***SE*** | ***95% CI*** | ***p*** |
| Age 🡪 maladaptive emotion regulation 🡪 T2 affect | .008 | .008 | −.008 – .023 | .328 | −.007 | .005 | −.017 – .002 | .111 |
| Age 🡪 adaptive emotion regulation 🡪 T2 affect | −.004 | .005 | −.013 – .005 | .335 | <.001 | .003 | −.006 – .005 | .870 |
| Age 🡪 mental flexibility 🡪 T2 affect | .009 | .007 | −.006 – .023 | .231 | −.006 | .003 | −.012 – .001 | .084 |

*Note.* Age and positive/negative affect were modelled as continuous observed variables and emotion regulation strategies and mental flexibility were modelled as latent variables. The maladaptive emotion regulation latent variable comprises bespoke items indexing frequency of use of maladaptive strategies. The adapative emotion regulation latent variable comprises bespoke items indexing frequency of use of adaptive strategies. The mental flexibility latent variable comprises items from the MFQ (Parsons et al., 2022). Positive affect reflects the average of positive emotions (i.e., content, happy, relieved, calm, appreciative) and negative affect reflects the average of negative emotions (i.e., anxious, angry, afraid, sad, worried, irritable, concerned, stressed, distressed, lonely, bored, hopeless, frustrated, disappointed) experienced in the previous two weeks because of the COVID–19 outbreak and resulting changes to daily life. T1 affect, gender, ethnicity, country, and COVID-19 risk were included as covariates. Paths included standardized *β*s, standardized SEs, and standardized 95% confidence intervals.

**SM Table 8**

*Direct Effects in the Relationship Between Age, Emotion Regulation Strategies, Mental Flexibility, Change in Affect from T1 to T3, and T3 Mental Health Problems (With Covariates)*

|  | **Positive Affect** | | | | **Negative Affect** | | | |
| --- | --- | --- | --- | --- | --- | --- | --- | --- |
|  | ***β*** | ***SE*** | ***95% CI*** | ***p*** | ***β*** | ***SE*** | ***95% CI*** | ***p*** |
| Maladaptive Emotion Regulation |  |  |  |  |  |  |  |  |
| ~Age | **−.061** | **.024** | **−.108 – −.013** | **.012** | **−.061** | **.024** | **−.108 – −.013** | **.013** |
| ~Female | −.022 | .021 | −.063 – .019 | .294 | −.022 | .021 | −.063 – .019 | .291 |
| ~White | **−.053** | **.023** | **−.098 – −.009** | **.019** | **−.054** | **.023** | **−.098 – −.009** | **.019** |
| ~UK | −.048 | .028 | −.102 – .007 | .088 | −.048 | .028 | −.103 – .007 | .088 |
| ~US | .001 | .028 | −.053 – .055 | .967 | .001 | .028 | −.053 – .055 | .974 |
| ~COVID-19 Risk | −.019 | .022 | −.061 – .023 | .377 | −.018 | .022 | −.061 – .024 | .392 |
| ~T1 Mental Health Problems | **.618** | **.025** | **.570 – .666** | **<.001** | **.618** | **.024** | **.570 – .666** | **<.001** |
| Adaptive Emotion Regulation |  |  |  |  |  |  |  |  |
| ~Age | **−.118** | **.031** | **−.178 – −.057** | **<.001** | **−.118** | **.031** | **−.179 – −.057** | **<.001** |
| ~Female | **.075** | **.029** | **.018 – .132** | **.010** | **.075** | **.029** | **.018 – .132** | **.010** |
| ~White | −.029 | .028 | −.084 – .026 | .302 | −.028 | .028 | −.083 – .026 | .309 |
| ~UK | **−.171** | **.032** | **−.234 – −.109** | **<.001** | **−.171** | **.032** | **−.234 – −.109** | **<.001** |
| ~US | −.039 | .031 | −.101 – .022 | .208 | −.040 | .031 | −.101 – .021 | .200 |
| ~COVID-19 Risk | **.062** | **.025** | **.012 – .112** | **.015** | **.062** | **.025** | **.012 – .112** | **.015** |
| ~T1 Mental Health Problems | **−.226** | **.037** | **−.298 – −.153** | **<.001** | **−.225** | **.037** | **−.297 – −.152** | **<.001** |
| Mental Flexibility |  |  |  |  |  |  |  |  |
| ~Age | .016 | .016 | −.016 – .048 | .330 | .016 | .016 | −.016 – .048 | .319 |
| ~Female | −.002 | .016 | −.034 – .031 | .927 | −.001 | .016 | −.034 – .031 | .933 |
| ~White | −.037 | .018 | −.073 – −.002 | .041 | −.038 | .018 | −.073 – −.002 | .038 |
| ~UK | **−.061** | **.020** | **−.100 – −.022** | **.002** | **−.060** | **.020** | **−.100 – −.021** | **.002** |
| ~US | .001 | .02 | −.038 – .041 | .941 | .002 | .020 | −.037 – .042 | .903 |
| ~COVID-19 Risk | .011 | .016 | −.020 – .043 | .487 | .011 | .016 | −.021 – .042 | .515 |
| ~T1 Mental Health Problems | **−.773** | **.013** | **−.799 – −.747** | **<.001** | **−.773** | **.013** | **−.799 – −.747** | **<.001** |
| Change in Affect |  |  |  |  |  |  |  |  |
| ~Maladaptive Emotion Regulation | <−.001 | .075 | −.146 – .146 | .997 | .130 | .075 | −.016 – .276 | .081 |
| ~Adaptive Emotion Regulation | .059 | .066 | −.069 – .188 | .366 | **−.191** | **.059** | **−.307 – −.075** | **.001** |
| ~Mental Flexibility | −.084 | .074 | −.228 – .060 | .254 | **.212** | **.073** | **.068 – .355** | **.004** |
| ~Age | **.102** | **.036** | **.032 – .172** | **.004** | .001 | .032 | −.061 – .063 | .968 |
| ~Female | −.060 | .038 | −.135 – .014 | .114 | .029 | .032 | −.033 – .091 | .363 |
| ~White | −.034 | .039 | −.110 – .042 | .380 | .021 | .035 | −.048 – .090 | .546 |
| ~UK | −.105 | .049 | −.201 – −.009 | .032 | **.305** | **.045** | **.218 – .392** | **<.001** |
| ~US | .007 | .045 | −.082 – .096 | .878 | **.325** | **.041** | **.245 – .404** | **<.001** |
| ~COVID-19 Risk | .023 | .032 | −.040 – .086 | .473 | −.041 | .032 | −.106 – .025 | .222 |
| ~T1 Mental Health Problems | .091 | .086 | −.078 – .259 | .292 | −.137 | .088 | −.309 – .035 | .118 |
| T3 Mental Health Problems |  |  |  |  |  |  |  |  |
| ~Maladaptive Emotion Regulation | .073 | .047 | −.019 – .166 | .121 | .039 | .042 | −.044 – .122 | .360 |
| ~Adaptive Emotion Regulation | **−.090** | **.037** | **−.163 – −.017** | **.016** | −.038 | .033 | −.103 – .026 | .246 |
| ~Mental Flexibility | .079 | .054 | −.026 – .185 | .141 | .020 | .052 | −.083 – .122 | .709 |
| ~Change in Affect | **−.237** | **.034** | **−.303 – −.171** | **<.001** | **.331** | **.042** | **.249 – .412** | **<.001** |
| ~Age | **−.084** | **.024** | **−.131 – −.036** | **.001** | **−.100** | **.022** | **−.143 – −.058** | **<.001** |
| ~Female | −.016 | .021 | −.057 – .026 | .464 | −.010 | .020 | −.049 – .030 | .633 |
| ~White | .060 | .029 | .003 – .116 | .038 | .060 | .027 | .007 – .112 | .025 |
| ~UK | .010 | .033 | −.054 – .074 | .754 | −.066 | .030 | −.124 – −.008 | .027 |
| ~US | **.080** | **.030** | **.021 – 138** | **.008** | −.027 | .028 | −.082 – .027 | .326 |
| ~COVID-19 Risk | −.026 | .021 | −.068 – .016 | .218 | −.015 | .017 | −.049 – .020 | .400 |
| ~T1 Mental Health Problems | **.825** | **.058** | **.710 – .939** | **<.001** | **.839** | **.061** | **.720 – .957** | **<.001** |

*Note*. Age and positive/negative affect were modelled as continuous observed variables and emotion regulation strategies were modelled as latent variables. The maladaptive emotion regulation latent variable comprises bespoke items indexing frequency of use of maladaptive strategies. The adapative emotion regulation latent variable comprises bespoke items indexing frequency of use of adaptive strategies. The mental flexibility latent variable comprises items from the MFQ (Parsons et al., 2022). Mental health problems were modelled as a higher order latent variable, as in Minihan et al. (2022), comprising depression symptoms measured with the PHQ–8 (Kroenke et al., 2001), anxiety symptoms measured with the GAD–7 (Spitzer et al., 2006), and mental wellbeing, measured with the 7–item WEMWBS (Stewart–Brown et al., 2009). Positive affect reflects the average of positive emotions (i.e., content, happy, relieved, calm, appreciative) and negative affect reflects the average of negative emotions (i.e., anxious, angry, afraid, sad, worried, irritable, concerned, stressed, distressed, lonely, bored, hopeless, frustrated, disappointed) experienced in the previous two weeks because of the COVID–19 outbreak and resulting changes to daily life. For gender, ‘other’ is the reference group, which includes responses options ‘male’ and ‘other’. For ethnicity, ‘other’ is the reference group, which includes responses options: ‘Asian’, ‘Hispanic’, ‘Black’, ‘Aboriginal or Torres Strait Islander’, ‘Mixed’, or ‘other’. For country, Australia is the reference group. COVID-19 risk was measured with a series of bespoke items indexing quarantining, diagnosis, hospitalization and death, described further in SM1. Paths included standardized *β*s, standardized SEs, and standardized 95% confidence intervals.

**SM Table 9**

*Direct Effects in the Relationship Between Age, Emotion Regulation Strategies, Mental Flexibility, Change in Affect from T1 to T3, and T3 Mental Health Problems (Without Covariates)*

|  | **Positive Affect** | | | | **Negative Affect** | | | |
| --- | --- | --- | --- | --- | --- | --- | --- | --- |
|  | ***β*** | ***SE*** | ***95% CI*** | ***p*** | ***β*** | ***SE*** | ***95% CI*** | ***p*** |
| Maladaptive Emotion Regulation |  |  |  |  |  |  |  |  |
| ~Age | **−.074** | **.024** | **−.121** – **−.027** | **.002** | **−.074** | **.024** | **−.121** – **−.027** | **.002** |
| ~T1 Mental Health Problems | **.610** | **.025** | **.561** – **.658** | **<.001** | **.610** | **.025** | **.562** – **.659** | **<.001** |
| Adaptive Emotion Regulation |  |  |  |  |  |  |  |  |
| ~Age | **−.114** | **.031** | **−.174 – −.053** | **<.001** | **−.114** | **.031** | **−.174 – −.053** | **<.001** |
| ~T1 Mental Health Problems | **−.241** | **.037** | **−.313 – −.170** | **<.001** | **−.240** | **.037** | **−.312 – −.169** | **<.001** |
| Mental Flexibility |  |  |  |  |  |  |  |  |
| ~Age | .010 | .016 | −.022 – .042 | .528 | .010 | .016 | −.021 – .042 | .523 |
| ~T1 Mental Health Problems | **−.773** | **.013** | **−.800 – −.747** | **<.001** | **−.773** | **.013** | **−.799 – −.747** | **<.001** |
| Change in Affect |  |  |  |  |  |  |  |  |
| ~Maladaptive Emotion Regulation | .009 | .074 | −.136 – .153 | .908 | .136 | .075 | −.011 – .284 | .070 |
| ~Adaptive Emotion Regulation | .072 | .065 | −.055 – .198 | .269 | **−.217** | **.059** | **−.333 – −.101** | **<.001** |
| ~Mental Flexibility | −.071 | .074 | −.216 – .074 | .338 | **.221** | **.076** | **.072 – .371** | **.004** |
| ~Age | **.094** | **.036** | **.024 – .164** | **.008** | .001 | .033 | −.063 – .065 | .980 |
| ~T1 Mental Health Problems | .094 | .085 | −.072 – .260 | .267 | −.098 | .088 | −.270 – .074 | .265 |
| T3 Mental Health Problems |  |  |  |  |  |  |  |  |
| ~Maladaptive Emotion Regulation | .069 | .046 | −.021 – .159 | .131 | .033 | .042 | −.049 – .114 | .428 |
| ~Adaptive Emotion Regulation | **−.091** | **.037** | **−.163 – −.018** | **.014** | −.035 | .033 | −.099 – .030 | .293 |
| ~Mental Flexibility | .076 | .056 | −.034 – .185 | .176 | .011 | .054 | −.095 – .116 | .843 |
| ~Change in Affect | **−.232** | **.033** | **−.297 – −.168** | **<.001** | **.319** | **.040** | **.240 – .397** | **<.001** |
| ~Age | **−.081** | **.024** | **−.129 – −.034** | **.001** | **−.094** | **.021** | **−.136 – −.053** | **<.001** |
| ~T1 Mental Health Problems | **.819** | **.058** | **.705 – .934** | **<.001** | **.818** | **.060** | **.700 – .936** | **<.001** |

*Note*. Age and positive/negative affect were modelled as continuous observed variables and emotion regulation strategies and mental flexibility were modelled as latent variables. The maladaptive emotion regulation latent variable comprises bespoke items indexing frequency of use of maladaptive strategies. The adapative emotion regulation latent variable comprises bespoke items indexing frequency of use of adaptive strategies. The mental flexibility latent variable comprises items from the MFQ (Parsons et al., 2022). Mental health problems were modelled as a higher order latent variable, as in Minihan et al. (2022), comprising depression symptoms measured with the PHQ–8 (Kroenke et al., 2001), anxiety symptoms measured with the GAD–7 (Spitzer et al., 2006), and mental wellbeing, measured with the 7–item WEMWBS (Stewart–Brown et al., 2009). Positive affect reflects the average of positive emotions (i.e., content, happy, relieved, calm, appreciative) and negative affect reflects the average of negative emotions (i.e., anxious, angry, afraid, sad, worried, irritable, concerned, stressed, distressed, lonely, bored, hopeless, frustrated, disappointed) experienced in the previous two weeks because of the COVID–19 outbreak and resulting changes to daily life. Paths included standardized *β*s, standardized SEs, and standardized 95% confidence intervals.

**SM Table 10**

*Indirect Effects in the Relationship Between Age, Emotion Regulation Strategies, Mental Flexibility, Change in Affect from T1 to T3, and T3 Mental Health Problems (Without Covariates)*

|  | **Positive Affect** | | | | **Negative Affect** | | | |
| --- | --- | --- | --- | --- | --- | --- | --- | --- |
|  | ***β*** | ***SE*** | ***95% CI*** | ***p*** | ***β*** | ***SE*** | ***95% CI*** | ***p*** |
| Age 🡪 Maladaptive Emotion Regulation 🡪 Change in Affect 🡪 T3 Mental Health Problems | <.001 | .001 | −.002 – .003 | .907 | −.003 | .002 | −.008 – .001 | .141 |
| Age 🡪 Adaptive Emotion Regulation 🡪 Change in Affect 🡪 T3 Mental Health Problems | .002 | .002 | −.002 – .005 | .296 | **.008** | **.003** | **.002 – .014** | **.014** |
| Age 🡪 Mental Flexibility 🡪 Change in Affect 🡪 T3 Mental Health Problems | <.001 | <.001 | <.001 – .001 | .600 | .001 | .001 | −.002 – .003 | .532 |
| Age 🡪 Maladaptive Emotion Regulation 🡪 T3 Mental Health Problems | −.005 | .004 | −.013 – .002 | .182 | −.002 | .003 | −.009 – .004 | .449 |
| Age 🡪 Adaptive Emotion Regulation 🡪 T3 Mental Health Problems | .010 | .005 | <.001 – .020 | .042 | .004 | .004 | −.004 – .012 | .309 |
| Age 🡪 Mental Flexibility 🡪 T3 Mental Health Problems | .001 | .001 | −.002 – .003 | .555 | <.001 | .001 | −.001 – .001 | .847 |
| Age 🡪 Change in Affect 🡪 T3 Mental Health Problems | **−.022** | **.009** | **−.039 – −.005** | **.013** | <.001 | .010 | −.020 – .021 | .980 |

*Note*. Age and positive/negative affect were modelled as continuous observed variables and emotion regulation strategies were modelled as latent variables. The maladaptive emotion regulation latent variable comprises bespoke items indexing frequency of use of maladaptive strategies. The adapative emotion regulation latent variable comprises bespoke items indexing frequency of use of adaptive strategies. The mental flexibility latent variable comprises items from the MFQ (Parsons et al., 2022). Mental health problems were modelled as a higher order latent variable, as in Minihan et al. (2022), comprising depression symptoms measured with the PHQ–8 (Kroenke et al., 2001), anxiety symptoms measured with the GAD–7 (Spitzer et al., 2006), and mental wellbeing, measured with the 7–item WEMWBS (Stewart–Brown et al., 2009). Positive affect reflects the average of positive emotions (i.e., content, happy, relieved, calm, appreciative) and negative affect reflects the average of negative emotions (i.e., anxious, angry, afraid, sad, worried, irritable, concerned, stressed, distressed, lonely, bored, hopeless, frustrated, disappointed) experienced in the previous two weeks because of the COVID–19 outbreak and resulting changes to daily life. T1 mental health problems were included as a covariate. Paths included standardized *β*s, standardized SEs, and standardized 95% confidence intervals.

**SM Table 11**

*Direct Effects in the Relationship Between Age, Emotion Regulation Strategies, Mental Flexibility, Change in Affect from T1 to T2, and T2 Mental Health Problems (With Covariates)*

|  | **Positive Affect** | | | | **Negative Affect** | | | |
| --- | --- | --- | --- | --- | --- | --- | --- | --- |
|  | ***β*** | ***SE*** | ***95% CI*** | ***p*** | ***β*** | ***SE*** | ***95% CI*** | ***p*** |
| Maladaptive emotion regulation |  |  |  |  |  |  |  |  |
| ~Age | **−.061** | **.024** | **−.108 – −.014** | **.013** | **−.061** | **.024** | **−.109 – −.014** | **.012** |
| ~Female | −.022 | .021 | −.063 – .018 | .280 | −.022 | .021 | −.063 – .019 | .286 |
| ~White | **−.053** | **.023** | **−.098 – −.008** | **.020** | **−.053** | **.023** | **−.098 – −.009** | **.019** |
| ~UK | −.049 | .028 | −.104 – .005 | .078 | −.049 | .028 | −.103 – .006 | .080 |
| ~US | <.001 | .028 | −.054 – .054 | .996 | <.001 | .028 | −.054 – .054 | .989 |
| ~COVID-19 Risk | −.018 | .021 | −.060 – .024 | .391 | −.019 | .021 | −.061 – .023 | .381 |
| ~T1 Mental Health Problems | **.618** | **.025** | **.569** – **.666** | **<.001** | **.617** | **.025** | **.568** – **.666** | **<.001** |
| Adaptive Emotion Regulation |  |  |  |  |  |  |  |  |
| ~Age | **−.117** | **.031** | **−.178 – −.056** | **<.001** | **−.117** | **.031** | **−.178 – −.056** | **<.001** |
| ~Female | **.075** | **.029** | **.018** – **.132** | **.010** | **.075** | **.029** | **.018** – **.132** | **.010** |
| ~White | −.029 | .028 | −.083 – .026 | .307 | −.029 | .028 | −.083 – .026 | .306 |
| ~UK | **−.171** | **.032** | **−.233 – −.108** | **<.001** | **−.171** | **.032** | **−.233 – −.108** | **<.001** |
| ~US | −.039 | .031 | −.100 – .022 | .210 | −.039 | .031 | −.100 – .022 | .211 |
| ~COVID-19 Risk | **.062** | **.025** | **.012** – **.112** | **.015** | **.062** | **.025** | **.012** – **.112** | **.015** |
| ~T1 Mental Health Problems | **−.224** | **.037** | **−.297 – −.152** | **<.001** | **−.224** | **.037** | **−.296 – −.151** | **<.001** |
| Mental Flexibility |  |  |  |  |  |  |  |  |
| ~Age | .016 | .016 | −.016 – .048 | .323 | .016 | .016 | −.016 – .049 | .317 |
| ~Female | −.001 | .016 | −.034 – .031 | .939 | −.001 | .016 | −.033 – .031 | .949 |
| ~White | −.037 | .018 | −.073 – −.002 | .041 | −.037 | .018 | −.073 – −.002 | .039 |
| ~UK | **−.060** | **.020** | **−.099 – −.021** | **.003** | **−.060** | **.020** | **−.099 – −.021** | **.003** |
| ~US | .002 | .020 | −.037 – .041 | .916 | .003 | .020 | −.037 – .042 | .899 |
| ~COVID-19 Risk | .011 | .016 | −.021 – .043 | .499 | .011 | .016 | −.021 – .043 | .497 |
| ~T1 Mental Health Problems | **−.773** | **.013** | **−.799 – −.747** | **<.001** | **−.773** | **.013** | **−.799 – −.746** | **<.001** |
| Change in Affect |  |  |  |  |  |  |  |  |
| ~Maladaptive Emotion Regulation | −.106 | .064 | −.231 – .019 | .097 | . 094 | .066 | −.035 – .222 | .154 |
| ~Adaptive Emotion Regulation | .026 | .057 | −.085 – .137 | .646 | −.089 | .051 | −.188 – .010 | .079 |
| ~Mental Flexibility | −.070 | .071 | −.210 – .070 | .326 | −.009 | .075 | −.156 – .138 | .904 |
| ~Age | .042 | .033 | −.022 – .107 | .196 | −.005 | .034 | −.071 – .061 | .875 |
| ~Female | **−.090** | **.032** | **−.152 – −.028** | **.004** | −.014 | .032 | −.076 – .049 | .667 |
| ~White | −.051 | .037 | −.123 – .022 | .170 | −.007 | .036 | −.078 – .064 | .840 |
| ~UK | .038 | .045 | −.049 – .126 | .391 | −.066 | .046 | −.156 – .023 | .146 |
| ~US | .082 | .041 | .002 – .163 | .045 | −.001 | .041 | −.082 – .080 | .984 |
| ~COVID-19 Risk | .009 | .026 | −.042 – .059 | .737 | −.026 | .028 | −.081 – .030 | .368 |
| ~T1 Mental Health Problems | **.176** | **.074** | **.032 – .320** | **.017** | **−.276** | **.081** | **−.435 – −.116** | **.001** |
| T2 Mental Health Problems |  |  |  |  |  |  |  |  |
| ~Maladaptive Emotion Regulation | .010 | .046 | −.080 – .100 | .826 | −.008 | .038 | −.083 – .067 | .829 |
| ~Adaptive Emotion Regulation | −.055 | .033 | −.120 – .010 | .099 | −.026 | .029 | −.083 – .032 | .386 |
| ~Mental Flexibility | −.042 | .051 | −.141 – .057 | .410 | −.031 | .044 | −.118 – .055 | .478 |
| ~Change in Affect | **−.156** | **.026** | **−.208 – −.105** | **<.001** | **.364** | **.028** | **.309 – .418** | **<.001** |
| ~Age | **−.053** | **.023** | **−.098 – −.008** | **.021** | **−.056** | **.021** | **−.097 – −.016** | **.006** |
| ~Female | −.031 | .021 | −.071 – .010 | .135 | −.013 | .017 | −.046 – .020 | .439 |
| ~White | −.021 | .024 | −.068 – .026 | .371 | −.017 | .021 | −.059 – .025 | .431 |
| ~UK | .024 | .028 | −.031 – .078 | .397 | .041 | .024 | −.007 – .089 | .094 |
| ~US | .018 | .026 | −.034 – .069 | .496 | −.006 | .023 | −.039 – .050 | .809 |
| ~COVID-19 Risk | −.017 | .023 | −.062 – .029 | .473 | −.014 | .022 | −.058 – .030 | .535 |
| ~T1 Mental Health Problems | **.783** | **.052** | **.681 – .886** | **<.001** | **.851** | **.048** | **.758 – .945** | **<.001** |

*Note*. Age and positive/negative affect were modelled as continuous observed variables and emotion regulation strategies were modelled as latent variables. The maladaptive emotion regulation latent variable comprises bespoke items indexing frequency of use of maladaptive strategies. The adapative emotion regulation latent variable comprises bespoke items indexing frequency of use of adaptive strategies. The mental flexibility latent variable comprises items from the MFQ (Parsons et al., 2022). Mental health problems were modelled as a higher order latent variable, as in Minihan et al. (2022), comprising depression symptoms measured with the PHQ–8 (Kroenke et al., 2001), anxiety symptoms measured with the GAD–7 (Spitzer et al., 2006), and mental wellbeing, measured with the 7–item WEMWBS (Stewart–Brown et al., 2009). Positive affect reflects the average of positive emotions (i.e., content, happy, relieved, calm, appreciative) and negative affect reflects the average of negative emotions (i.e., anxious, angry, afraid, sad, worried, irritable, concerned, stressed, distressed, lonely, bored, hopeless, frustrated, disappointed) experienced in the previous two weeks because of the COVID–19 outbreak and resulting changes to daily life. For gender, ‘other’ is the reference group, which includes responses options ‘male’ and ‘other’. For ethnicity, ‘other’ is the reference group, which includes responses options: ‘Asian’, ‘Hispanic’, ‘Black’, ‘Aboriginal or Torres Strait Islander’, ‘Mixed’, or ‘other’. For country, Australia is the reference group. COVID-19 risk was measured with a series of bespoke items indexing quarantining, diagnosis, hospitalization and death, described further in SM1. Paths included standardized *β*s, standardized SEs, and standardized 95% confidence intervals.

**SM Table 12**

*Indirect Effects in the Relationship Between Age, Emotion Regulation Strategies, Mental Flexibility, Change in Affect from T1 to T2, and T2 Mental Health Problems (With Covariates)*

|  | **Positive Affect** | | | | **Negative Affect** | | | |
| --- | --- | --- | --- | --- | --- | --- | --- | --- |
|  | ***β*** | ***SE*** | ***95% CI*** | ***p*** | ***β*** | ***SE*** | ***95% CI*** | ***p*** |
| Age 🡪 maladaptive emotion regulation 🡪 change in affect 🡪 T2 mental health problems | −.001 | .001 | −.003 – .001 | .210 | −.002 | .002 | −.005 – .001 | .229 |
| Age 🡪 adaptive emotion regulation 🡪 change in affect 🡪 T2 mental health problems | <.001 | .001 | −.002 – .003 | .654 | .004 | .002 | −.001 – .009 | .124 |
| Age 🡪 mental flexibility 🡪 change in affect 🡪 T2 mental health problems | <.001 | <.001 | <.001 – .001 | .487 | <.001 | <.001 | −.001 – .001 | .904 |
| Age 🡪 maladaptive emotion regulation 🡪 T2 mental health problems | −.001 | .003 | −.006 – .005 | .826 | .001 | .002 | −.004 – .005 | .830 |
| Age 🡪 adaptive emotion regulation 🡪 T2 mental health problems | .006 | .004 | −.002 – .015 | .129 | .003 | .004 | −.004 – .010 | .397 |
| Age 🡪 mental flexibility 🡪 T2 mental health problems | −.001 | .001 | −.003 – .001 | .535 | −.001 | .001 | −.002 – .001 | .569 |
| Age 🡪 change in affect 🡪 T2 mental health problems | −.007 | .005 | −.017 – .004 | .208 | −.002 | .012 | −.026 – .022 | .875 |

*Note*. Age and positive/negative affect were modelled as continuous observed variables and emotion regulation strategies were modelled as latent variables. The maladaptive emotion regulation latent variable comprises bespoke items indexing frequency of use of maladaptive strategies. The adapative emotion regulation latent variable comprises bespoke items indexing frequency of use of adaptive strategies. The mental flexibility latent variable comprises items from the MFQ (Parsons et al., 2022). Mental health problems were modelled as a higher order latent variable, as in Minihan et al. (2022), comprising depression symptoms measured with the PHQ–8 (Kroenke et al., 2001), anxiety symptoms measured with the GAD–7 (Spitzer et al., 2006), and mental wellbeing, measured with the 7–item WEMWBS (Stewart–Brown et al., 2009). Positive affect reflects the average of positive emotions (i.e., content, happy, relieved, calm, appreciative) and negative affect reflects the average of negative emotions (i.e., anxious, angry, afraid, sad, worried, irritable, concerned, stressed, distressed, lonely, bored, hopeless, frustrated, disappointed) experienced in the previous two weeks because of the COVID–19 outbreak and resulting changes to daily life. T1 mental health problems, gender, ethnicity, country, and COVID-19 risk were included as covariates. Paths included standardized *β*s, standardized SEs, and standardized 95% confidence intervals.

**SM Table 13**

*Analyses as per Pre–Registration: Direct Effects in the Relationship Between Age, Emotion Regulatory Processes, and T3 Affect (With Covariates)*

|  | **Positive Affect** | | | | **Negative Affect** | | | |
| --- | --- | --- | --- | --- | --- | --- | --- | --- |
|  | ***β*** | ***SE*** | ***95% CI*** | ***p*** | ***β*** | ***SE*** | ***95% CI*** | ***p*** |
| Maladaptive Emotion Regulation |  |  |  |  |  |  |  |  |
| ~Age | **−.234** | **.020** | **−.273 – −.196** | **<.001** | **−.149** | **.018** | **−.184 – −.114** | **<.001** |
| ~Female | **.093** | **.020** | **.054 – .133** | **<.001** | **.059** | **.017** | **.026 – .091** | **<.001** |
| ~White | −.009 | .020 | −.049 – .031 | .659 | .032 | .018 | −.003 – .067 | .071 |
| ~UK | .054 | .024 | .006 – .102 | .028 | −.044 | .022 | −.087 – −.001 | .045 |
| ~US | .005 | .024 | −.042 – .052 | .841 | **−.091** | **.021** | **−.133 – −.049** | **<.001** |
| ~COVID-19 Risk | .038 | .021 | −.003 – .079 | .067 | .002 | .018 | −.033 – .037 | .907 |
| ~T1 Affect | **−.335** | **.020** | **−.374 – −.296** | **<.001** | **.593** | **.015** | **.563 – .623** | **<.001** |
| Adaptive Emotion Regulation |  |  |  |  |  |  |  |  |
| ~Age | **−.091** | **.032** | **−.153 – −.029** | **.004** | **−.078** | **.032** | **−.140 – −.016** | **.014** |
| ~Female | **.068** | **.029** | **.012** – **.125** | **.018** | **.076** | **.029** | **.019** – **.132** | **.008** |
| ~White | −.011 | .028 | −.066 – .044 | .692 | −.027 | .028 | −.082 – .029 | .351 |
| ~UK | **−.177** | **.032** | **−.239 – −.115** | **<.001** | **−.189** | **.032** | **−.253 – −.125** | **<.001** |
| ~US | −.039 | .031 | −.101 – .022 | .212 | −.047 | .032 | −.109 – .016 | .143 |
| ~COVID-19 Risk | .053 | .026 | .003 – .103 | .039 | .055 | .026 | .004 – .106 | .035 |
| ~T1 Affect | **.205** | **.029** | **.149** – **.262** | **<.001** | −.058 | .033 | −.123 – .008 | .084 |
| Mental Flexibility |  |  |  |  |  |  |  |  |
| ~Age | **.139** | **.019** | **.101** – **.177** | **<.001** | **.070** | **.018** | **.034** – **.106** | **<.001** |
| ~Female | −.025 | .021 | −.065 – .016 | .231 | .013 | .018 | −.023 – .049 | .486 |
| ~White | .003 | .021 | −.037 – .043 | .887 | **−.048** | **.021** | **−.089 – −.007** | **.020** |
| ~UK | **−.109** | **.024** | **−.156 – −.062** | **<.001** | −.025 | .023 | −.069 – .019 | .264 |
| ~US | −.016 | .024 | −.063 – .031 | .511 | **.070** | **.023** | **.025** – **.114** | **.002** |
| ~COVID-19 Risk | −.018 | .020 | −.057 – .022 | .376 | .017 | .019 | −.020 – .055 | .368 |
| ~T1 Affect | **.476** | **.018** | **.441** – **.511** | **<.001** | **−.605** | **.017** | **−.637 – −.572** | **<.001** |
| T3 Affect |  |  |  |  |  |  |  |  |
| ~Maladaptive Emotion Regulation | **−.127** | **.043** | **−.212 – −.042** | **.003** | **.194** | **.038** | **.120** – **.268** | **<.001** |
| ~Adaptive Emotion Regulation | .115 | .052 | .012 – .217 | .028 | −.075 | .040 | −.153 – .003 | .059 |
| ~Mental Flexibility | <.001 | .054 | −.106 – .106 | .999 | .046 | .045 | −.043 – .134 | .309 |
| ~Age | **.099** | **.030** | **.039** – **.158** | **.001** | −.010 | .023 | −.054 – .035 | .674 |
| ~Female | −.014 | .031 | −.075 – .048 | .659 | .011 | .023 | −.035 – .057 | .637 |
| ~White | −.060 | .031 | −.120 – <.001 | .051 | −.005 | .024 | −.053 – .042 | .826 |
| ~UK | **−.134** | **.042** | **−.216 – −.051** | **.001** | **.298** | **.033** | **.234** – **.363** | **<.001** |
| ~US | −.061 | .039 | −.137 – .014 | .112 | **.310** | **.030** | **.251** – **.370** | **<.001** |
| ~COVID-19 Risk | .012 | .028 | −.042 – .067 | .652 | −.027 | .024 | −.074 – .020 | .264 |
| ~T1 Affect | **.432** | **.034** | **.366** – **.498** | **<.001** | **.545** | **.039** | **.469** – **.621** | **<.001** |

*Note.* Age and positive/negative affect were modelled as continuous observed variables and emotion regulation strategies and mental flexibility were modelled as latent variables. The maladaptive emotion regulation latent variable comprises items from the PSWQ-C (Chorpita et al., 1997) and bespoke items indexing frequency of use of maladaptive strategies. The adapative emotion regulation latent variable comprises bespoke items indexing frequency of use of adaptive strategies. The mental flexibility latent variable comprises items from the MFQ (Parsons et al., 2022). Positive affect reflects the average of positive emotions (i.e., content, happy, relieved, calm, appreciative) and negative affect reflects the average of negative emotions (i.e., anxious, angry, afraid, sad, worried, irritable, concerned, stressed, distressed, lonely, bored, hopeless, frustrated, disappointed) experienced in the previous two weeks because of the COVID–19 outbreak and resulting changes to daily life. For gender, ‘other’ is the reference group, which includes responses options ‘male’ and ‘other’. For ethnicity, ‘other’ is the reference group, which includes responses options: ‘Asian’, ‘Hispanic’, ‘Black’, ‘Aboriginal or Torres Strait Islander’, ‘Mixed’, or ‘other’. For country, Australia is the reference group. COVID-19 risk was measured with a series of bespoke items indexing quarantining, diagnosis, hospitalization and death, described further in SM1. Paths included standardized *β*s, standardized SEs, and standardized 95% confidence intervals.

**SM Table 14**

*Analyses as per Pre–Registration: Indirect Effects in the Relationship Between Age, Emotion Regulatory Processes, and T3 Affect (With Covariates)*

|  | **Positive Affect** | | | | **Negative Affect** | | | |
| --- | --- | --- | --- | --- | --- | --- | --- | --- |
|  | ***β*** | ***SE*** | ***95% CI*** | ***p*** | ***β*** | ***SE*** | ***95% CI*** | ***p*** |
| Age 🡪 Maladaptive Emotion Regulation 🡪 T3 Affect | **.030** | **.010** | **.009 – .050** | **.004** | **−.029** | **.007** | **−.042 – −.016** | **<.001** |
| Age 🡪 Adaptive Emotion Regulation 🡪 T3 Affect | −.010 | .006 | −.022 – .001 | .072 | .006 | .004 | −.002 – .013 | .132 |
| Age 🡪 Mental Flexibility 🡪 T3 Affect | <.001 | .008 | −.015 – .015 | .999 | .003 | .003 | −.003 – .010 | .324 |

*Note.* Age and positive/negative affect were modelled as continuous observed variables and emotion regulatory processes were modelled as latent variables, indexing maladaptive emotion regulation, adaptive emotion regulation, and mental flexibility. The maladaptive emotion regulation latent variable comprises items from the PSWQ-C (Chorpita et al., 1997) and bespoke items indexing frequency of use of maladaptive strategies. The adapative emotion regulation latent variable comprises bespoke items indexing frequency of use of adaptive strategies. The mental flexibility latent variable comprises items from the MFQ (Parsons et al., 2022). Positive affect reflects the average of positive emotions (i.e., content, happy, relieved, calm, appreciative) and negative affect reflects the average of negative emotions (i.e., anxious, angry, afraid, sad, worried, irritable, concerned, stressed, distressed, lonely, bored, hopeless, frustrated, disappointed) experienced in the previous two weeks because of the COVID-19 outbreak and resulting changes to daily life. T1 affect, gender, ethnicity, country, and COVID-19 risk were included as covariates. Paths included standardized *β*s, standardized SEs, and standardized 95% confidence intervals.

**SM Table 15**

*Analyses as Per Pre–Registration: Direct Effects in the Relationship Between Age, Emotion Regulatory Processes Change in Affect from T1 to T3, and T3 Mental Health Problems*

|  | **Positive Affect** | | | | **Negative Affect** | | | |
| --- | --- | --- | --- | --- | --- | --- | --- | --- |
|  | ***β*** | ***SE*** | ***95% CI*** | ***p*** | ***β*** | ***SE*** | ***95% CI*** | ***p*** |
| Maladaptive Emotion Regulation |  |  |  |  |  |  |  |  |
| ~Age | **−.093** | **.017** | **−.125 – −.061** | **<.001** | **−.093** | **.017** | **−.125 – −.061** | **<.001** |
| ~Female | **.070** | **.014** | **.042 – .098** | **<.001** | **.070** | **.014** | **.042 – .098** | **<.001** |
| ~White | .022 | .015 | −.008 – .052 | .155 | .022 | .015 | −.008 – .052 | .154 |
| ~UK | −.009 | .018 | −.045 – .027 | .622 | −.009 | .018 | −.045 – .027 | .624 |
| ~US | −.029 | .018 | −.064 – .006 | .101 | −.029 | .018 | −.064 – .006 | .102 |
| ~COVID-19 Risk | .008 | .016 | −.024 – .040 | .612 | .008 | .016 | −.024 – .040 | .628 |
| ~T1 Mental Health Problems | **.786** | **.012** | **.763 – .810** | **<.001** | **.787** | **.012** | **.763 – .810** | **<.001** |
| Adaptive Emotion Regulation |  |  |  |  |  |  |  |  |
| ~Age | **−.114** | **.031** | **−.175 – −.053** | **<.001** | **−.114** | **.031** | **−.175 – −.053** | **<.001** |
| ~Female | **.077** | **.029** | **.020 – .134** | **.008** | **.077** | **.029** | **.020 – .134** | **.008** |
| ~White | −.028 | .028 | −.083 – .027 | .323 | −.027 | .028 | −.082 – .028 | .331 |
| ~UK | **−.174** | **.032** | **−.237 – −.111** | **<.001** | **−.174** | **.032** | **−.237 – −.112** | **<.001** |
| ~US | −.041 | .031 | −.103 – .020 | .189 | −.042 | .031 | −.103 – .020 | .182 |
| ~COVID-19 Risk | **.060** | **.026** | **.010 – .110** | **.018** | **.060** | **.026** | **.010 – .110** | **.018** |
| ~T1 Mental Health Problems | **−.212** | **.036** | **−.283 – −.142** | **<.001** | **−.211** | **.036** | **−.282 – −.140** | **<.001** |
| Mental Flexibility |  |  |  |  |  |  |  |  |
| ~Age | .018 | .016 | −.014 – .050 | .268 | .019 | .016 | −.014 – .051 | .257 |
| ~Female | .002 | .017 | −.031 – .034 | .910 | .002 | .017 | −.031 – .035 | .902 |
| ~White | −.037 | .018 | −.073 – −.001 | .042 | −.038 | .018 | −.074 – −.002 | .039 |
| ~UK | **−.064** | **.020** | **−.104 – −.025** | **.001** | **−.064** | **.020** | **−.103 – −.025** | **.001** |
| ~US | .003 | .020 | −.036 – .043 | .864 | .004 | .020 | −.035 – .044 | .826 |
| ~COVID-19 Risk | .011 | .016 | −.021 – .043 | .495 | .010 | .016 | −.021 – .042 | .524 |
| ~T1 Mental Health Problems | **−.767** | **.014** | **−.794 – −.740** | **<.001** | **−.767** | **.014** | **−.793 – −.740** | **<.001** |
| Change in Affect |  |  |  |  |  |  |  |  |
| ~Maladaptive Emotion Regulation | **−.244** | **.065** | **−.372 – −.117** | **<.001** | **.207** | **.065** | **.079 – .336** | **.002** |
| ~Adaptive Emotion Regulation | .080 | .061 | −.041 – .200 | .194 | **−.184** | **.055** | **−.291 – −.077** | **.001** |
| ~Mental Flexibility | −.109 | .073 | −.252 – .034 | .134 | **.205** | **.071** | **.066 – .344** | **.004** |
| ~Age | .080 | .036 | .010 – .150 | .025 | .015 | .032 | −.046 – .077 | .624 |
| ~Female | −.048 | .037 | −.121 – .026 | .204 | .014 | .032 | −.050 – .077 | .676 |
| ~White | −.034 | .039 | −.110 – .042 | .383 | .010 | .035 | −.059 – .078 | .777 |
| ~UK | **−.111** | **.049** | **−.206 – −.016** | **.022** | **.311** | **.044** | **.224 – .398** | **<.001** |
| ~US | .002 | .045 | −.086 – .090 | .963 | **.332** | **.040** | **.254 – .411** | **<.001** |
| ~COVID-19 Risk | .026 | .032 | −.037 – .089 | .417 | −.048 | .032 | −.111 – .015 | .139 |
| ~T1 Mental Health Problems | **.262** | **.086** | **.094 – .430** | **.002** | **−.228** | **.090** | **−.405 – −.052** | **.011** |
| T3 Mental Health Problems |  |  |  |  |  |  |  |  |
| ~Maladaptive Emotion Regulation | .071 | .054 | −.035 – .178 | .188 | .068 | .051 | −.033 – .168 | .188 |
| ~Adaptive Emotion Regulation | **−.087** | **.037** | **−.159 – −.015** | **.017** | −.041 | .032 | −.104 – .022 | .201 |
| ~Mental Flexibility | .058 | .052 | −.044 – .159 | .265 | .004 | .050 | −.094 – .103 | .934 |
| ~Change in Affect | **−.230** | **.035** | **−.299 – −.160** | **<.001** | **.332** | **.043** | **.247 – .418** | **<.001** |
| ~Age | **−.080** | **.024** | **−.128 – −.032** | **.001** | **−.095** | **.021** | **−.137 – −.053** | **<.001** |
| ~Female | −.020 | .022 | −.063 – .022 | .347 | −.013 | .020 | −.053 – .026 | .508 |
| ~White | .053 | .029 | −.005 – .110 | .071 | .056 | .027 | .003 – .108 | .039 |
| ~UK | .018 | .033 | −.046 – .083 | .577 | −.059 | .030 | −.118 – <.001 | .050 |
| ~US | **.085** | **.030** | **.027 – .144** | **.004** | −.023 | .028 | −.079 – .032 | .408 |
| ~COVID-19 Risk | −.031 | .021 | −.072 – .010 | .137 | −.018 | .018 | −.052 – .016 | .302 |
| ~T1 Mental Health Problems | **.793** | **.070** | **.656 – .930** | **<.001** | **.796** | **.071** | **.657 – .936** | **<.001** |

*Note.* Age and positive/negative affect were modelled as continuous observed variables and emotion regulatory processes were modelled as latent variables, indexing maladaptive emotion regulation, adaptive emotion regulation, and mental flexibility. The maladaptive emotion regulation latent variable comprises items from the PSWQ–C (Chorpita et al., 1997) and bespoke items indexing frequency of use of maladaptive strategies. The adapative emotion regulation latent variable comprises bespoke items indexing frequency of use of adaptive strategies. The mental flexibility latent variable comprises items from the MFQ (Parsons et al., 2022). Mental health problems were modelled as a higher order latent variable, as in Minihan et al. (2022), comprising depression symptoms measured with the PHQ–8 (Kroenke et al., 2001), anxiety symptoms measured with the GAD–7 (Spitzer et al., 2006), and mental wellbeing, measured with the 7–item WEMWBS (Stewart–Brown et al., 2009). Positive affect reflects the average of positive emotions (i.e., content, happy, relieved, calm, appreciative) and negative affect reflects the average of negative emotions (i.e., anxious, angry, afraid, sad, worried, irritable, concerned, stressed, distressed, lonely, bored, hopeless, frustrated, disappointed) experienced in the previous two weeks because of the COVID–19 outbreak and resulting changes to daily life. For gender, ‘other’ is the reference group, which includes responses options ‘male’ and ‘other’. For ethnicity, ‘other’ is the reference group, which includes responses options: ‘Asian’, ‘Hispanic’, ‘Black’, ‘Aboriginal or Torres Strait Islander’, ‘Mixed’, or ‘other’. For country, Australia is the reference group. COVID–19 risk was measured with a series of bespoke items indexing quarantining, diagnosis, hospitalization and death, described further in SM1. Paths included standardized *β*s, standardized SEs, and standardized 95% confidence intervals.

**SM Table 16**

*Analyses as Per Pre–Registration: Indirect Effects in the Relationship Between Age, Emotion Regulatory Processes Change in Affect from T1 to T3, and T3 Mental Health Problems*

|  | **Positive Affect** | | | | **Negative Affect** | | | |
| --- | --- | --- | --- | --- | --- | --- | --- | --- |
|  | ***β*** | ***SE*** | **95% CI** | ***p*** | ***β*** | ***SE*** | ***95% CI*** | ***p*** |
| Age 🡪 Maladaptive Emotion Regulation 🡪 Change in Affect 🡪 T3 Mental Health Problems | **−.005** | **.002** | **−.009 – −.002** | **.004** | **−.006** | **.003** | **−.011 – −.001** | **.011** |
| Age 🡪 Adaptive Emotion Regulation 🡪 Change in Affect 🡪 T3 Mental Health Problems | .002 | .002 | −.001 – .006 | .231 | **.007** | **.003** | **.001 – .013** | **.022** |
| Age 🡪 Mental Flexibility 🡪 Change in Affect 🡪 T3 Mental Health Problems | <.001 | .001 | −.001 – .001 | .373 | .001 | .001 | −.001 – .004 | .288 |
| Age 🡪 Maladaptive Emotion Regulation 🡪 T3 Mental Health Problems | −.007 | .005 | −.017 – .004 | .204 | −.006 | .005 | −.016 – .003 | 0.203 |
| Age 🡪 Adaptive Emotion Regulation 🡪 T3 Mental Health Problems | .010 | .005 | <.001 – .020 | .047 | .005 | .004 | −.003 – .012 | .225 |
| Age 🡪 Mental Flexibility 🡪 T3 Mental Health Problems | .001 | .001 | −.001 – .004 | .414 | <.001 | .001 | −.002 – .002 | .933 |

| Age 🡪 Change in Affect 🡪 T3 Mental Health Problems | −.018 | .009 | −.035 – −.001 | .034 | .005 | .010 | −.015 – .026 | .622 |
| --- | --- | --- | --- | --- | --- | --- | --- | --- |

*Note.* Age and positive/negative affect were modelled as continuous observed variables and emotion regulatory processes were modelled as latent variables, indexing maladaptive emotion regulation, adaptive emotion regulation, and mental flexibility. The maladaptive emotion regulation latent variable comprises items from the PSWQ-C (Chorpita et al., 1997) and bespoke items indexing frequency of use of maladaptive strategies. The adapative emotion regulation latent variable comprises bespoke items indexing frequency of use of adaptive strategies. The mental flexibility latent variable comprises items from the MFQ (Parsons et al., 2022). Mental health problems were modelled as a higher order latent variable, as in Minihan et al. (2022), comprising depression symptoms measured with the PHQ-8 (Kroenke et al., 2001), anxiety symptoms measured with the GAD-7 (Spitzer et al., 2006), and mental wellbeing, measured with the 7-item WEMWBS (Stewart-Brown et al., 2009). Positive affect reflects the average of positive emotions (i.e., content, happy, relieved, calm, appreciative) and negative affect reflects the average of negative emotions (i.e., anxious, angry, afraid, sad, worried, irritable, concerned, stressed, distressed, lonely, bored, hopeless, frustrated, disappointed) experienced in the previous two weeks because of the COVID-19 outbreak and resulting changes to daily life. T1 mental health problems, gender, ethnicity, country, and COVID-19 risk were included as covariates. Paths included standardized *β*s, standardized SEs, and standardized 95% confidence intervals.
